# Brief alcohol exposure alters transcription in astrocytes via the heat shock pathway

**DOI:** 10.1002/brb3.125

**Published:** 2013-02-06

**Authors:** Leonardo Pignataro, Florence P Varodayan, Lindsay E Tannenholz, Petr Protiva, Neil L Harrison

**Affiliations:** 1 Department of Anesthesiology The College of Physicians and Surgeons, Columbia University630 West 168th St., New York, NY, 10032; 2 Department of Neuroscience The College of Physicians and Surgeons, Columbia University630 West 168th St., New York, NY, 10032; 3 Department of Pharmacology The College of Physicians and Surgeons, Columbia University630 West 168th St., New York, NY, 10032; 4 Veteran's Affairs Medical CenterWest Haven, CT, 06516; 5 MYSM School Of Medicine, Yale University15 York St., New Haven, CT, 06510

**Keywords:** Alcohol, alcohol response element, astrocytes, gene expression, glia, heat shock factor 1, microarray

## Abstract

Astrocytes are critical for maintaining homeostasis in the central nervous system (CNS), and also participate in the genomic response of the brain to drugs of abuse, including alcohol. In this study, we investigated ethanol regulation of gene expression in astrocytes. A microarray screen revealed that a brief exposure of cortical astrocytes to ethanol increased the expression of a large number of genes. Among the alcohol-responsive genes (ARGs) are glial-specific immune response genes, as well as genes involved in the regulation of transcription, cell proliferation, and differentiation, and genes of the cytoskeleton and extracellular matrix. Genes involved in metabolism were also upregulated by alcohol exposure, including genes associated with oxidoreductase activity, insulin-like growth factor signaling, acetyl-CoA, and lipid metabolism. Previous microarray studies performed on ethanol-treated hepatocyte cultures and mouse liver tissue revealed the induction of almost identical classes of genes to those identified in our microarray experiments, suggesting that alcohol induces similar signaling mechanisms in the brain and liver. We found that acute ethanol exposure activated heat shock factor 1 (HSF1) in astrocytes, as demonstrated by the translocation of this transcription factor to the nucleus and the induction of a family of known HSF1-dependent genes, the heat shock proteins (*Hsps*). Transfection of a constitutively transcriptionally active *Hsf1* construct into astrocytes induced many of the ARGs identified in our microarray study supporting the hypothesis that HSF1 transcriptional activity, as part of the heat shock cascade, may mediate the ethanol induction of these genes. These data indicate that acute ethanol exposure alters gene expression in astrocytes, in part via the activation of HSF1 and the heat shock cascade.

## Introduction

Astrocytes are the most abundant cell type in the central nervous system (CNS). They were originally regarded as passive structural elements that provided a substrate for neuronal growth and synaptic connectivity. More recent work suggests a prominent and active role for astrocytes in maintaining homeostasis in the CNS. Astrocytes remove and recycle neurotransmitters and ions from the synaptic cleft (Vernadakis 1988; Wang and Bordey 2008), regulate local pH (Belanger and Magistretti 2009), and protect neurons from reactive oxygen species (ROS) that are generated as a consequence of the high metabolic rate of brain tissue (Aschner and Kimelberg 1991; Kirchhoff et al. 2001; Gonzalez and Salido 2009). Astrocytes also contribute to the CNS immunological response as they synthesize cytokines, including tumor necrosis factor-α (TNF-α), interleukin-1β (IL-1β), IL-6, IL-10, IL-15, interferon β (INFβ), and transforming growth factor-β (TGF-β) (Tacconi 1998; Norenberg 2005; Farina et al. 2007).

Changes in the expression of the astrocytic marker glial fibrillary acidic protein (GFAP) occur after administration of alcohol and other drugs of abuse, demonstrating that astrocytes are targeted by these substances (Stiene-Martin et al. 1991; Franke 1995; Guerri and Renau-Piqueras 1997; Valles et al. 1997; Fattore et al. 2002; Gonca et al. 2005; Dalcik et al. 2009b). Despite this evidence, little is known about the role of astrocytes in the brain's adaptative response to drugs of abuse (Miguel-Hidalgo 2009). Recent studies that begin to address this question suggest that astrocyte activity is necessary for the expression of the rewarding effects of morphine and methamphetamine in the mouse and for the development of tolerance to these drugs (Song and Zhao 2001; Narita et al. 2006, 2008). Therefore, it appears that astrocytes actively participate in the integrated response of the brain to drugs of abuse.

In the case of alcohol, several microarray studies of postmortem frontal cortex tissue from alcoholic patients have found altered expression of astrocyte-specific genes (Liu et al. 2007) and genes generally associated with glial function (Mayfield et al. 2002). This important and clinically relevant evidence suggests that astrocytes contribute to the global response of the human brain to alcohol exposure by altering their patterns of gene expression. Despite these indications, there has been no comprehensive global analysis of alcohol-induced gene expression changes specifically in astrocytes, and the mechanisms by which ethanol modulates the regulation of genes in these cells remain unknown. Most of the previous work on the effects of alcohol on glial gene expression has been performed using postmortem brain samples from human alcoholics (Mayfield et al. 2002; Liu et al. 2007) and interpretation of these results is difficult due to the cellular heterogeneity of the tissue and uncertainty regarding the drug history of the subjects. In this study, we have investigated the effects of acute application of ethanol on a pure astrocyte population under controlled in vitro conditions, in order to probe potential mechanisms for alcohol effects on gene expression. We have identified a large number of important gene clusters and some novel individual alcohol-responsive genes (ARGs), while also uncovering an underlying regulatory mechanism by which alcohol triggers an early adaptive genomic response in these cells.

## Materials and Methods

### Cell culture and immunocytochemistry

Primary cultures of cortical astrocytes were prepared from embryonic day 17–18 C57/BL6 mice according to the standard Banker and Goslin's (1998) technique with modifications (Ma et al. 2004). Cells were plated at a density of 0.3 × 10^6^ cells/mL on precoated 0.05 mg/mL poly-d-lysine (Sigma, St. Louis, MO) plates, and maintained in minimal Eagle's medium (MEM; Gibco, Grand Island, NY) supplemented with 10% vol/vol horse serum (Sigma) and 0.5 mmol/L l-glutamine (Gibco). The low plating density and medium changes every other day reduced neuronal survival close to zero, while sustaining an almost pure population of astrocytes. Experiments were carried out no sooner than 14 days after plating to ensure the development of a mature astrocyte population in the cultures.

Immunostaining was done as previously described (Pignataro et al. 2007). The antibodies used were affinity-purified rabbit anti-HSF1 antibody (0.08 μg/mL, Cell Signaling Technology, Danvers, MA) and guinea pig polyclonal anti-human GFAP antibody (5 μg/mL, Synaptic Systems, Goettingen, Germany). Cells were mounted with ProLong Gold anti-fade reagent containing the nuclear stain 4′,6-diamidino-2-phenylindole (DAPI, Molecular Probes, Grand Island, NY). To determine the purity of the cultures, cells were also stained with isolectin IB_4_ from *Griffonia simplicifolia* (50 μg/mL, Molecular Probes) and rabbit polyclonal antiserum against coronin-1a (Novus, 1/200 dilution; Littleton, CO) that specifically label microglial cells (Chung and Han 2003; Ahmed et al. 2007). Images were acquired with an inverted Zeiss Axiovert 200 confocal microscope (LSM 510 META; Carl Zeiss Microimaging Inc., Thornwood, NY) equipped with diode (405 nm), argon (458, 477, 488, and 514 nm), HeNe1 (543 nm), and HeNe2 (633 nm) lasers.

### Ethanol and heat shock treatment

When primary astrocytes were almost completely confluent (DIV14 onwards), cultures were exposed to ethanol or heat for specific time periods (1 h for RNA experiments or 2 h to determine changes in protein expression). Ethanol (absolute, 200 proof, Sigma) was added directly to the culture medium to achieve a final concentration of 60 mmol/L. We have previously used this ethanol concentration and exposure time without significant consequences on cell survival (Pignataro et al. 2007). Control cells received vehicle (phosphate buffered saline or medium). Cells were subjected to heat shock by transferring them to an incubator set at 42°C for a period of 1–2 h.

### Gene arrays

For gene microarray analysis, total RNA was isolated from control cells or from cells treated with alcohol or heat. Five hundred nanograms of total RNA was used to make biotin-labeled cRNA using the Illumina total RNA amplification and labeling kit (Ambion, Grand Island, NY). Biotinylated cRNA was labeled with fluorescent dye at the Rockefeller University Gene Array Facility, hybridized onto a MouseRef-8 v2.0 Expression BeadChip expression array (Illumina, San Diego, CA) and scanned. Arrays were normalized by shift to 75th percentile and expression values below noise level were set to the minimum detection level. Expression data were then analyzed by Genespring software (Agilent Technologies, Santa Clara, CA). Quality control was performed by analyzing gene expression correlation coefficients and samples with coefficients less than 0.95 were excluded. There were duplicate control samples, triplicate ethanol-treated samples, and duplicate heat-treated samples with correlation coefficients of >0.99 between biological replicates. For the array analysis, biological replicate sample signals were averaged. The differences in gene expression were determined using analysis of variance (ANOVA) post hoc adjusted by Tukey test (*P* < 0.05), and multiple hypothesis testing adjustments were made using the Benjamini–Hochberg method at a false discovery rate (FDR) of less than 0.05.

For gene array analysis, a hierarchical clustering algorithm was used to generate the dendrogram based on the squared Euclidian distance method with complete-linkage (Eisen et al. 1998). Genes differentially expressed following ethanol or heat treatments were subjected to Gene Ontology (GO) enrichment analysis using the hypergeometric method corrected by Benjamini–Yekuteili method at FDR ≤0.25.

**Figure 1:**
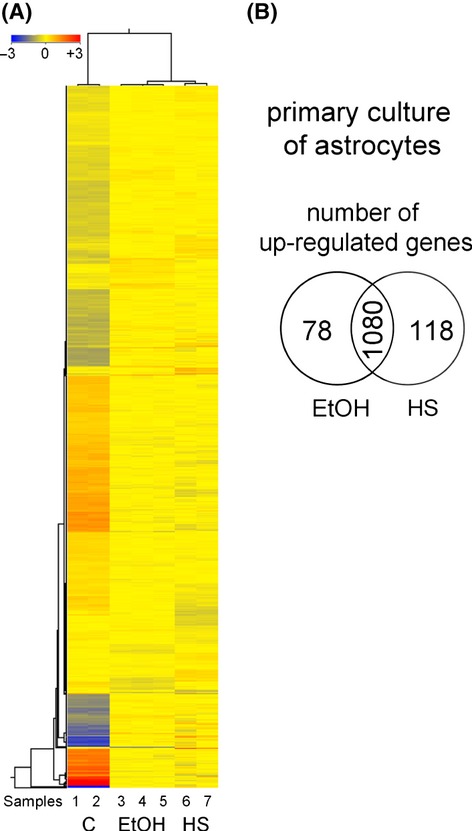
Hierarchical clustering by squared Euclidean distance algorithm on differentially expressed genes and Venn diagram of ethanol- and heat-induced genes in primary astrocyte culture. (A) The graph shows hierarchical clustering of the gene expression pattern after treatment as analyzed by squared Euclidean distance algorithm on differentially expressed genes detected by adjusted ANOVA test (post hoc adjusted by Tukey test, *P* < 0.05, FDR <0.05). Samples were treated for 1 h with 60 mmol/L ethanol (EtOH) or 42°C heat stress (HS). The columns represent the individual samples and the color scale on top represents the log transformed relative change in expression (red indicates gene induction and blue downregulation of genes). The samples grouped according to treatment and EtOH-induced genes largely overlapped with those induced by HS. (B) Venn diagram of significant EtOH- and HS-induced genes versus controls in primary culture of astrocytes. The intersection denotes the number of genes responsive to both treatments.

**Figure 2:**
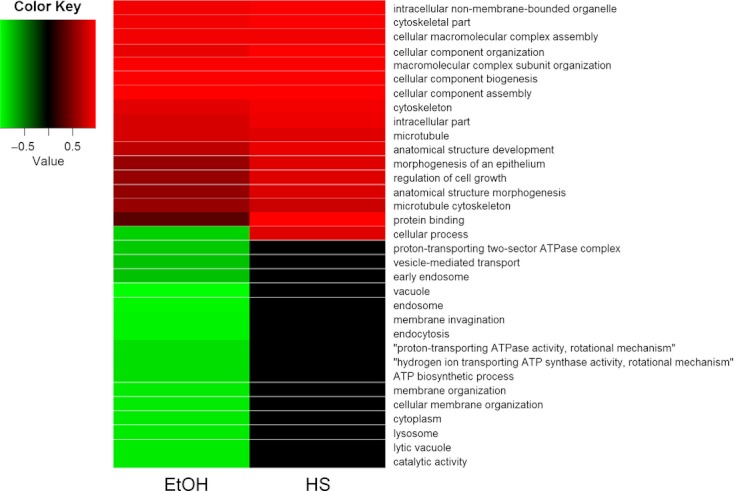
Heat-map of Gene Ontology categories enrichment analysis across the ethanol (EtOH) and heat shock (HS) treatments. Only categories with an adjusted FDR-q-value of less than 0.25 in at least one condition are shown in the figure. Colors indicate downregulation (green) or upregulation (red), and values = 1 − FDR-q (with downregulation given negative values). Note that genes induced by EtOH or HS treatments tend to belong to the same GO categories while the enrichment results are more heterogeneous among the downregulated genes.

In order to identify genes regulated by both ethanol and heat shock in astrocytes, we analyzed the results of the microarray experiments looking for genes induced by both treatments. There was a substantial overlap between the transcriptional profiles of the two treatments, suggesting similar mechanisms of gene regulation (Fig. 1). Comparison of GO enrichment using differentially expressed genes after ethanol or heat treatment also showed striking similarities among upregulated categories (Fig. 2).

### Real-time polymerase chain reaction analyses of mRNA levels

Total RNA was isolated from cultured cells using TRIzol (Invitrogen, Grand Island, NY). cDNA was prepared from total RNA with the iScript cDNA synthesis kit (Bio-Rad, Hercules, CA). For cDNA preparation, reactions were performed in a final volume of 20 μL; primers were annealed at 25°C for 5 min and RNA was reverse transcribed at 42°C for 90 min, followed by RNAse H digestion. Enzymes were subsequently heat inactivated at 95°C for 5 min and the reaction mixtures were stored at −20°C. The first strand reverse transcribed cDNA was used as a template for polymerase chain reaction (PCR) amplification using the appropriate specific primer pairs listed below. Quantitative “real-time” reverse transcriptase PCR (Q-PCR) was carried out as previously described (Ma et al. 2004). For each sample, the cDNA concentration for the gene of interest was normalized against the concentration of *Actb* and *Rn18S* (rRNA 18S gene; QuantumRNA Internal Standards, Ambion) cDNA in the same sample, and the results were finally expressed as a percentage of increase above the control (untreated cells or cells treated with vehicle). As there was no significant difference between the data normalized with the two housekeeping genes (Fig. S2), subsequent experiments were normalized with *Actb* cDNA. For each experiment, the average values of triplicate samples from several independent experiments were used for each data point, as indicated in the figure legend. A control sample in which reverse transcriptase was omitted from the reaction was included in each experiment to monitor for genomic DNA contamination.

### Q-PCR primers

The following primers were used in the Q-PCR reactions:

*Acas21*: forward (5′-GTTTGGGACACTCCTTACCATAC-3′), reverse (5′-AGGCAGTTGACAGACACATTC-3′);
*Acot11*: forward (5′-AGGGGCTTCGCCTCTATGTT-3′), reverse (5′-TCCGGTATCCTTCACCCTCTG-3′);
*Acta2*: forward (5′-GTCCCAGACATCAGGGAGTAA-3′), reverse (5′-TCGGATACTTCAGCGTCAGGA-3′);
*Actb*: forward (5′-TCATGAAGTGTGACGTTGACATCCGT-3′), reverse (5′-CCTAGAAGCATTTGCGGTGCACGATG-3′);
*Aldh1l1*: forward (5′-CAGGAGGTTTACTGCCAGCTA-3′), reverse (5′-CACGTTGAGTTCTGCACCCA-3′);
*Cryab*: forward (5′-GAAGAACGCCAGGACGAACAT-3′), reverse (5′-ACAGGGATGAAGTGATGGTGAG-3′);
*Ctgf*: forward (5′-AAGGGCCTCTTCTGCGATTTC-3′), reverse (5′-TGCACACTCCGATCTTGCG-3′);
*Gapdh*: forward (5′-AACTTTGGCATTGTGGAAGG-3′), reverse (5′-ACACATTGGGGGTAGGAACA-3′);
*Gas6*: forward (5′-CCGCGCCTACCAAGTCTTC-3′), reverse (5′-CGGGGTCGTTCTCGAACAC-3′);
*Hsp27*: forward (5′-ATCCCCTGAGGGCACACTTA-3′), reverse (5′-CCAGACTGTTCAGACTTCCCAG-3′);
*Hsp40*: forward (5′-TTCGACCGCTATGGAGAGGAA-3′), reverse (5′-CACCGAAGAACTCAGCAAACA-3′);
*Hsp70*: forward (5′-AATTGGCTGTATGAAGATGG-3′), reverse (5′-CATTGGTGCTTTTCTCTACC-3′);
*Hsp90*: forward (5′-GAACATTGTGAAGAAGTGCC-3′), reverse (5′-CATATACACCACCTCGAAGC-3′);
*Hsp110*: forward (5′-CAGGTACAAACTGATGGTCAACA-3′), reverse (5′-TGAGGTAAGTTCAGGTGAAGGG-3′);
*Igfbpl1*: forward (5′-GGGACTCAAGTATTCCTTTCCTG-3′), reverse (5′-GCACCTGGACAGCTATATTGAC-3′);
*Igfbp2*: forward (5′-CAGACGCTACGCTGCTATCC-3′), reverse (5′-CTCCCTCAGAGTGGTCGTCA-3′).

### Immunoblotting

The relative abundance of heat shock proteins (HSPs) was determined by immunoblotting, as previously described (Jia et al. 2005). Cellular fractions were isolated with the NE-PER Nuclear and Cytoplasmic Extraction Reagents (Pierce Biotechnology, Rockford, IL). Samples (40 μg of protein) were incubated with antibodies against αβ-crystallin (3.6 μg/mL, Novus), HSP27 (0.1 μg/mL, Invitrogen), HSP40 (0.12 μg/mL, Cell Signaling Technology), HSP70 (1 μg/mL, Invitrogen), HSP90 (0.03 μg/mL, Cell Signaling Technology), and HSP105/110 (2 μ/ml, Novus). To normalize for protein loading, transfer, and detection, the blots were also immunostained with antibodies against the translation initiation factor eIF4E (0.3 μg/mL, Cell Signaling Technology) or α-tubulin (0.47 μg/mL, Sigma). Images were acquired with a Biospectrum imaging system (UVP, Upland, CA) equipped with a refrigerated Chemi 410 CCD camera and the VisionWorks LS software (UVP). Digital images were quantified using Scion Image for Windows beta 4.0.2 (SCION Corp., Frederick, MD). Gel lanes were selected and the signals transformed into peaks. The area under each peak (gray value) was transformed into an optical density (OD) value using the function: OD = Log_10_ (255/[255 − gray value]). The OD values of the protein of interest were normalized to the eIF4E or α-tubulin internal standard to compensate for variations in protein loading and transfer.

### Analysis of colocalization of HSF1 and the nuclear stain DAPI

To investigate the possible translocation of HSF1 to the nucleus, astrocytes were immunostained with rabbit anti-HSF1 antibody and the cell nucleus was stained with DAPI. Confocal images were acquired, with care taken to avoid pixel saturation to prevent false colocalization. Gray scale 8-bit calibrated images (0.8–1 μm optical sections) were evaluated for colocalization of HSF1 and DAPI signals by a global statistic approach that performs intensity correlation coefficient–based analyses. We use the algorithm JACoP (Bolte and Cordelieres 2006) that calculates the Pearson's coefficient of pixel intensity in both channels represented in a scatter plot. The slope of the linear regression provides the rate of association of the signals ranging from 1 (total overlapping) to −1 (complete exclusion).

### Constitutively transcriptionally active *Hsf1* construct

We made use of a constitutively transcriptionally active form of HSF1 (*Hsf1*-act, BH-S) to determine whether the genes identified in the gene array are dependent on the activation of HSF1 for their expression. *Hsf1*-act has a long deletion of amino acids 203–315 in the regulatory domain of HSF1 (Zuo et al. 1995). The construct was generated by Dr. Richard Voellmy (University of Miami) and cloned into the pcDNA3.1^+^ vector (Invitrogen). Transfections were performed with 1.5 μg of DNA, 3 μL of lipofectamine LTX (Invitrogen), and 1.7 μL of Plus reagent (Invitrogen), and sister cultures were transfected with an empty pcDNA3.1^+^ vector as a control. Cells were used 24–48 h after transfection. Optimization of the transfection protocol and previous work from our laboratory demonstrated that this proportion of lipid/DNA results in a high efficiency of transfection, producing a significant expression of the construct of interest with the desired cellular phenotype (Fig. S3; Pignataro et al. 2007).

### Database search

For all genes analyzed, mouse genomic DNA sequences were obtained from the National Center for Biotechnology Information (NCBI; National Institutes of Health) and Mouse Genomic Informatics (Jackson Laboratory, Bar Harbor, ME) databases. DNA sequence analyses were performed using the Vector NTI program (Invitrogen). Candidate genes were designated as those containing one of more ARE core motifs, CTGNGTC, and at least eight matches of the 11 bp sequence of the complete ARE (TCTGCGTCTCT) anywhere between 0.5 kb upstream of the ATG or downstream in exon/intron region.

### Statistical analysis

Details of the statistical analysis and *P* values of the data are included in the figure legends, as appropriate. All data are presented as mean ± SEM. In all cases in which inmmunoblots or images are shown, the data are representative of at least three experiments with similar results.

### Supplemental data

Supplemental data are available as Supporting Information.

## Results

### Genome profiling of ARGs

In this study, we used primary cultures containing a relatively pure (≥90%) population of cortical mouse astrocytes to investigate the effects of a brief alcohol exposure on gene expression. Gene expression data were generated from ethanol-treated (60 mmol/L, 1 h) primary cultures that were >90% positive for the mature astrocytic marker GFAP. Immunocytochemical analysis of the cultures for the microglial markers coronin-1a and isolectin-B_4_ (Calka et al. 1996; Chung and Han 2003; Yokoyama et al. 2004; Yenari et al. 2006; Ahmed et al. 2007) revealed that the microglial contamination is less than ∼3% (Fig. S1). The ethanol concentration used in this study (60 mmol/L), although relatively high, is well within the range associated with human intoxication, as chronic alcoholics regularly sustain blood alcohol concentration levels between 50 and 100 mmol/L and often function normally when their levels exceed 100 mmol/L (Urso et al. 1981).

### Gene array analysis

Two thousand and four hundred transcripts were identified as differentially expressed across the treatment groups (using an adjusted ANOVA test at a corrected *P* level of ≤0.05). There was a substantial overlap of ∼85% between genes significantly expressed in response to heat shock or ethanol treatment, suggesting that the transcriptional response to ethanol resembles the heat shock response. We have reported similar findings in our previous work in neurons (Pignataro et al. 2007; Varodayan et al. 2011). Indeed, unsupervised hierarchical cluster analysis on genes and conditions clearly shows the high degree of similarity in gene expression patterns between the ethanol and heat treatments. The ethanol-treated samples cluster was distinct from the data for the heat shock samples, however, suggesting there are also some important differences between the glial responses to heat and ethanol (Fig. 1A).

Figure 2 shows that both ethanol and heat shock treatment significantly increased the expression of GO categories related to nonmembrane bound organelle, cytoskeleton, macromolecular assembly, cellular organization, microtubules, cell growth and protein binding, among others. This analysis revealed a high degree of similarity between the ethanol- and heat shock-induced processes and some heterogeneity among the downregulated processes, suggesting that the two treatments share some common mechanisms but do not operate via a single identical mechanism of gene regulation in astrocytes.

### Identification of ARGs containing the alcohol response element

We previously identified a novel mechanism for the ethanol induction of genes in cortical neurons, involving the binding of the activated form of the transcription factor heat shock factor 1 (HSF1) to an 11-bp DNA consensus sequence termed the alcohol response element (ARE; Pignataro et al. 2007). To determine whether ethanol regulates ARGs in astrocytes in a similar manner to that observed in neurons, we analyzed the results of the ethanol and heat stress microarrays to identify genes with a similar degree of induction by both treatments. One thousand and eighty unique genes were significantly upregulated to a similar magnitude by both treatments using a corrected *P* level of ≤0.05 (Fig. 1B and Table S1). Among this set of ethanol- and heat shock-sensitive genes, there were a variety of different functional gene groups: regulation of transcription, cell proliferation and differentiation, oxidoreductase activity, insulin-like growth factor signaling, calcium signaling, inflammatory/immune response, acetyl-CoA metabolism, serine/threonine kinase activity, cytoskeleton, lipid metabolism, apoptosis, glial-specific genes, and stress proteins (Table 1).

**Table 1:** Genes significantly activated by ethanol and heat stress in primary astrocyte culture

Gene	Accession number	*P*-value	CvsE	CvsHS	Definition
Regulation of transcription					
*Ddx3y*	NM_012008.1	0.009	6.35	5.59	DEAD (Asp-Glu-Ala-Asp) box polypeptide 3, Y-linked
*Olig1*	NM_016968.2	0.014	4.19	3.69	Oligodendrocyte transcription factor 1
*Idb4*	NM_031166.1	0.003	3.59	4.09	Inhibitor of DNA binding 4
*S100a1*	NM_011309.2	0.016	3.17	3.00	S100 calcium-binding protein A1
*Taf13*	NM_025444	0.003	2.35	2.47	TAF13 RNA polymerase II, TATA box-binding protein
*Ddx25*	NM_013932.2	0.015	2.31	1.98	DEAD (Asp-Glu-Ala-Asp) box polypeptide 25
*H2afx*	NM_010436.2	0.011	2.30	1.95	H2A histone family, member X
*Taf9b*	NM_001001176.1	0.013	2.15	1.78	TAF9B RNA polymerase II, TATA box-binding protein (TBP)-associated factor
*Hist1h2bh*	NM_178197.1	0.018	1.98	2.02	Histone cluster 1, H2bh
Cell proliferation and differentiation					
*Lgals3*	NM_010705.1	0.001	65.53	72.30	Lectin, galactose binding, soluble 3
***Igfbpl1***	**NM_018741.1**	**0.002**	**21.05**	**19.28**	**Insulin-like growth factor binding protein-like 1**
***Igfbp2***	**NM_008342.2**	**0.003**	**14.96**	**17.18**	**Insulin-like growth factor binding protein 2**
*Tst*	NM_009437.2	0.012	5.49	4.33	Thiosulfate sulfurtransferase, mitochondrial
*Shh*	NM_009170	0.018	5.46	6.94	Sonic hedgehog
*Dlk1*	NM_010052	0.005	5.01	5.76	Delta-like 1 homolog (*Drosophila*)
*Idb4*	NM_031166.1	0.003	3.59	4.09	Inhibitor of DNA binding 4
*Pdgfb*	NM_011057.2	0.002	3.38	4.36	Platelet-derived growth factor, B polypeptide
*Ptx3*	NM_008987.2	0.008	3.28	3.76	Pentraxin-related gene
*Meis1*	NM_010789.1	0.004	3.18	3.12	Myeloid ecotropic viral integration site 1
*Dlx2*	NM_010054.1	0.012	2.80	4.07	Distal-less homeobox 2
*Fgf13*	NM_010200.2	0.015	2.22	2.37	Fibroblast growth factor 13
*Gfap*	NM_010277	0.024	2.02	1.85	Glial fibrillary acidic protein
*Idb3*	NM_008321.1	0.017	1.77	2.59	Inhibitor of DNA binding 3
*Net1*	NM_019671	0.017	1.55	1.56	Neuroepithelial cell transforming gene 1
Oxidoreductase activity					
*Lox*	NM_010728.1	0.014	6.02	8.33	Lysyl oxidase
*Cyp1b1*	NM_009994	0.005	4.72	5.52	P450, family 1, subfamily b, polypeptide 1
*Tst*	NM_009437.2	0.012	5.49	4.33	Thiosulfate sulfurtransferase, mitochondrial (Tst), mRNA.
*Gstt3*	NM_133994.2	0.003	4.83	4.14	Glutathione *S*-transferase, theta 3
*Loxl1*	NM_010729	0.012	4.04	4.14	Lysyl oxidase-like 1
*Cp*	NM_007752.2	0.014	3.32	3.22	Ceruloplasmin
*Fthfd*	NM_027406.1	0.011	3.15	3.13	Aldehyde dehydrogenase 1 family, member L1
*Cyp4f14*	NM_022434.1	0.038	2.48	1.99	Cytochrome P450, family 4, subfamily f, polypeptide 14
*Coxvib2*	NM_183405.1	0.009	2.35	2.14	Cytochrome c oxidase subunit VIb polypeptide 2
*Gstk1*	NM_029555.2	0.013	1.95	1.49	Glutathione *S*-transferase kappa 1
*Nqo2*	NM_020282.2	0.035	1.87	1.85	NAD(P)H dehydrogenase, quinone 2
*Glrx*	NM_053108.2	0.036	1.56	1.38	Glutaredoxin
*Ndufc1*	NM_025523.1	0.035	1.39	1.57	NADH dehydrogenase (ubiquinone) 1, subcomplex unknown, 1
***Aldh1l1***	**NM_009656.1**	**0.007**	**1.46**	**1.49**	**Aldehyde dehydrogenase 1 family, member L1**
*Aldh5a1*	NM_172532	0.015	1.64	1.57	Aldhehyde dehydrogenase family 5, subfamily A1
Insulin-like growth factor signaling					
***Igfbpl1***	**NM_018741.1**	**0.002**	**21.05**	**19.28**	**Insulin-like growth factor binding protein-like 1**
***Igfbp2***	**NM_008342.2**	**0.003**	**14.96**	**17.18**	**Insulin-like growth factor binding protein 2**
***Ctgf***	**NM_010217.1**	**0.002**	**11.02**	**15.12**	**Connective tissue growth factor**
*Grb10*	NM_010345	0.002	5.18	6.48	Growth factor receptor bound protein 10
Calcium signaling					
*Fbln1*	NM_010180.1	0.007	11.22	11.17	Fibulin 1
*Dlk1*	NM_010052	0.005	5.01	5.76	Delta-like 1 homolog (*Drosophila*)
*Dlx2*	NM_010054.1	0.012	2.80	4.07	Distal-less homeobox 2
*Ednrb*	NM_007904.2	0.025	5.32	3.42	Endothelin receptor type B
*Scube3*	NM_001004366.1	0.006	3.03	2.76	Signal peptide, CUB domain, EGF-like 3
*Calm3*	NM_007590.2	0.019	1.49	1.37	Calmodulin 3
*Camk2 g*	NM_178597.2	0.020	1.25	1.38	Calcium/calmodulin-dependent protein kinase II gamma
Inflammatory/immune response					
*Entpd2*	NM_009849.1	0.012	8.03	6.27	Ectonucleoside triphosphate diphosphohydrolase 2
***Gas6***	**NM_019521.1**	**0.007**	**7.82**	**6.79**	**Growth arrest specific 6**
*Pea15*	NM_008556.1	0.003	6.32	6.23	Phosphoprotein enriched in astrocytes 15
*Rgs16*	NM_011267.1	0.015	5.68	10.14	Regulator of G-protein signaling 16
*Cmtm7*	NM_133978.1	0.005	5.01	5.03	CKLF-like MARVEL transmembrane domain containing 7
*Cd97*	NM_011925.1	0.005	3.18	3.49	CD97 antigen
*Insl6*	NM_013754.1	0.006	3.05	2.33	Insulin-like 6
*Cd59a*	NM_007652.2	0.005	2.90	2.31	CD59a antigen
*Fas*	NM_007987.1	0.001	2.80	2.59	Fas (TNF receptor superfamily member)
*Igsf9*	NM_033608.2	0.012	2.78	2.11	Immunoglobulin superfamily, member 9
*Igsf11*	NM_170599.2	0.005	2.60	1.89	Immunoglobulin superfamily, member 11
*Rgs7*	NM_011880.1	0.034	2.57	2.05	Regulator of G-protein signaling 7
*Mmd2*	NM_175217.3	0.035	2.51	1.84	Monocyte to macrophage differentiation associated 2
*Il18*	NM_008360.1	0.032	2.03	2.08	Interleukin 18
*Il6st*	NM_010560	0.048	1.93	1.98	Interleukin 6 signal transducer
*Il17d*	NM_145837.1	0.012	1.87	1.94	Interleukin 17D
*Il17rb*	NM_019583	0.037	1.68	1.58	Interleukin 17 receptor B
*Il7*	NM_008371.2	0.031	1.57	1.56	Interleukin 7
*Nfatc1*	NM_198429.1	0.016	1.54	1.25	Nuclear factor of activated T cells, cytoplasmic, calcineurin dependent 1
*Tnfaip2*	NM_009396.1	0.032	1.50	1.28	Tumor necrosis factor, alpha-induced protein 2
Acetyl-CoA metabolism					
***Acas2 l***	**NM_080575.1**	**0.013**	**4.89**	**4.63**	**Acyl-CoA synthetase short-chain family member 1**
***Acot11***	**NM_025590.3**	**0.019**	**4.03**	**3.07**	**Acyl-CoA thioesterase 11**
*Acot1*	NM_012006.2	0.014	3.92	2.78	Acyl-CoA thioesterase 1
*Nudt7*	NM_024437.1	0.007	3.20	2.60	Nudix (nucleoside diphosphate linked moiety X)-type motif 7
*Acsbg1*	NM_053178.1	0.009	2.47	2.32	Acyl-CoA synthetase bubblegum family member 1
*Ivd*	NM_019826	0.027	1.59	1.54	Isovaleryl-CoA dehydrogenase
*Acad9*	NM_172678.2	0.044	1.45	1.43	Acyl-CoA dehydrogenase family, member 9
Serine/threonine kinase activity					
*Ccnd1*	NM_007631.1	0.038	5.72	7.26	Cyclin D1
*Akap12*	NM_031185.1	0.007	3.64	4.92	A kinase (PRKA) anchor protein (gravin) 12
*Ephb2*	XM_204072.3	0.007	3.42	2.82	Eph receptor B2
*Ang1*	NM_007447.2	0.032	3.26	3.20	Angiogenin, ribonuclease, RNase A family, 5
*Prkcd*	NM_011103.1	0.004	2.26	2.04	Protein kinase C, delta
*Prkcdbp*	NM_028444.1	0.016	2.23	2.51	Protein kinase C, delta-binding protein
*Sgk3*	NM_177547.2	0.008	1.53	1.38	Serum/glucocorticoid-regulated kinase 3
*Prkra*	NM_011871.1	0.037	1.33	1.41	Protein kinase, interferon inducible double stranded RNA-dependent activator
Cytoskeleton					
*Tuba6*	XM_147357.1	0.010	6.96	6.39	Tubulin alpha 6
*Tpm2*	NM_009416.2	0.012	5.03	5.01	Tropomyosin 2, beta
***Acta2***	**NM_007392**	**0.009**	**3.38**	**3.44**	**Actin, alpha 2, smooth muscle, aorta**
*Actn4*	NM_021895.2	0.010	1.91	2.66	Actinin alpha 4
*Tubb2b*	NM_023716.1	0.012	1.61	1.38	Tubulin, beta 2b
*Acta1*	NM_009606.1	0.037	1.23	1.24	Actin, alpha 1, skeletal muscle
*Mark2*	NM_007928	0.037	1.15	1.20	MAP/microtubule affinity-regulating kinase 2
Lipid metabolism					
*Smpdl3a*	NM_020561.1	0.012	5.69	4.87	Sphingomyelin phosphodiesterase, acid-like 3A
*Mest*	NM_008590.1	0.013	4.35	4.68	Mesoderm-specific transcript
*Acot11*	NM_025590.3	0.019	4.03	3.07	Acyl-CoA thioesterase 11
*Lpl*	NM_008509.1	0.007	4.00	3.06	Lipoprotein lipase
*Acot1*	NM_012006.2	0.014	3.92	2.78	Acyl-CoA thioesterase 1
*Mgll*	NM_011844.3	0.031	2.98	2.24	Monoglyceride lipase
*Smpd3*	NM_021491.2	0.047	1.58	1.79	Sphingomyelin phosphodiesterase 3
Apoptosis					
*Col18a1*	NM_009929.2	0.003	9.98	12.86	Procollagen, type XVIII, alpha 1
*Gas6*	NM_019521.1	0.007	7.82	6.79	Growth arrest specific 6
*Pea15*	NM_008556.1	0.003	6.32	6.23	Phosphoprotein enriched in astrocytes 15
*Rtn1*	NM_153457.4	0.010	6.70	5.60	Reticulon 1
*Idb4*	NM_031166.1	0.003	3.59	4.09	Inhibitor of DNA binding 4
*Ddit4 l*	NM_030143.2	0.015	2.99	3.53	DNA-damage-inducible transcript 4-like
*Gadd45b*	NM_008655.1	0.022	1.86	2.76	Growth arrest and DNA-damage-inducible 45 beta
*Casp1*	NM_009807.1	0.004	2.38	1.77	Caspase 1
*Ddit4*	NM_029083.1	0.014	1.26	1.77	DNA-damage-inducible transcript 4
Glial-specific genes					
*Pea15*	NM_008556.1	0.003	6.32	6.23	Phosphoprotein enriched in astrocytes 15
*Olig1*	NM_016968.2	0.014	4.19	3.69	Oligodendrocyte transcription factor 1
*Metrn*	NM_133719	0.011	2.25	2.17	Meteorin, glial cell differentiation regulator
*Colm*	NM_177350.2	0.027	2.12	2.32	Gliomedin
*Gfap*	NM_010277	0.024	2.02	1.85	Glial fibrillary acidic protein
*Mbp*	NM_001025245.1	0.046	1.39	1.28	Myelin basic protein
*Nrcam*	NM_176930.2	0.023	1.20	1.21	Neuron-glia-CAM-related cell adhesion molecule
Stress proteins					
*Crygs*	NM_009967.1	0.004	9.34	10.30	Crystallin, gamma S
*Hrsp12*	NM_008287.2	0.027	1.58	1.35	Heat-responsive protein 12
*Dnajc7*	NM_019795.3	0.029	1.56	1.34	DnaJ (Hsp40) homolog, subfamily C, member 7
*Hspb1*	NM_013560	0.016	1.44	8.18	Heat shock protein 1
*Hspb8*	NM_030704.1	0.044	1.41	1.56	Heat shock protein 8
*Hspa5 bp1*	NM_133804.1	0.044	1.27	1.18	Heat shock protein 5 binding protein 1
*Hspa1a*	NM_010479.2	0.010	1.11	18.31	Heat shock protein 1A

Table of some of the genes significantly induced by 60 mmol/L ethanol (E) and 42°C heat stress (HS) for 1 h. Data were obtained by hybridization of seven biologically independent samples with treatments performed at least in duplicate. The differences in gene expressions were determined using ANOVA post hoc adjusted by Tukey test (*P* < 0.05) and the multiple hypothesis testing adjustments were made using the Benjamini–Hochberg method at a false discovery rate (FDR) of less than 0.05. The complete list of genes is presented in Table S1. Genes marked in bold were further characterized by Q-PCR and HSF1 construct transfection.

### Ethanol activates HSF1 and the expression of HSPs in astrocytes

The microarray analysis also revealed that ethanol treatment induced several genes encoding for HSPs (*Hsps*) (Table 1), including the gene homolog of *Hsp40* (*Dnajc7*) and members of the *Hsp27* family of HSP genes (*Hspb1* and *Hspb8*). In addition, ethanol upregulated the genes coding for the binding proteins *Hsp70* and *Hspa5 bp1*, as well as *Hspa1a*, which encodes the protein 1A of the *Hsp70* family.

**Figure 3:**
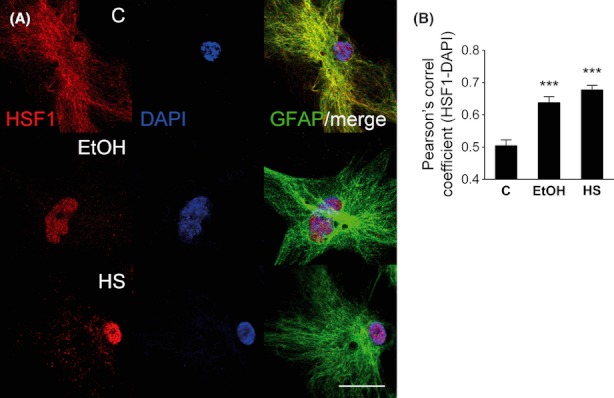
Ethanol induces heat shock factor 1 (HSF1) protein translocation into the nucleus of cortical astrocytes. (A) Ethanol (EtOH) and heat shock (HS) treatment caused the translocation of HSF1 into the nucleus of primary cultured astrocytes. Immunostaining was performed with an HSF1-specific antibody (red) and DAPI nuclear staining (blue). Cells were also positive for a marker of mature astrocytes, glial fibrillary acidic protein (GFAP; green). (B) Quantification was performed by Pearson's correlation coefficient of pixel intensity scatter plots. The colocalization of HSF1 and DAPI signals increases with EtOH and HS treatment of primary astrocytes. All data are the mean ± SEM of *n* ≥ 30 cells from two independent cultures and were compared with control by one-way ANOVA with Dunnett's multiple comparison post hoc test (significantly different at the level of ***P < 0.001). The scale bar represents 20 μm.

It is known that the induction of HSPs is dependent on the multi-step activation of HSF1. In unstressed cells, the chaperone proteins HSP40, HSP70, and HSP90 bind to HSF1, sequestering inactive HSF1 in the cytoplasm (Morimoto et al. 1998; Tonkiss and Calderwood 2005). Stress causes protein misfolding in the cytoplasm, which triggers the release of HSF1 from the chaperone HSPs, and allows its subsequent translocation into the cell nucleus (Morimoto et al. 1998). Once in the nucleus, HSF1 trimerizes and acquires DNA-binding properties. Following hyperphosphorylation, HSF1 becomes transcriptionally competent and is able to induce the expression of multiple *Hsp* genes and other stress-responsive genes. In order to determine whether the gene expression changes identified in the microarray study were dependent on the transcription factor HSF1, we first investigated whether ethanol treatment of astrocytes can activate HSF1 by promoting its translocation into the nucleus. Immunostaining of primary astrocytes with a HSF1-specific antibody and the nuclear stain DAPI showed that ethanol (60 mmol/L, 1 h) caused extensive translocation of HSF1 from the cytoplasm to the nucleus of the cells (Fig. 3A), similar to the effects of heat shock treatment. Quantification of the immunocytochemistry results obtained with the primary astrocytes indicated that the colocalization of HSF1 and DAPI (measured by Pearson's correlation coefficient between locations of these two markers) increased significantly upon exposure to ethanol or heat (Fig. 3B). The ability of ethanol to stimulate HSF1 nuclear translocation suggests that activation of this transcription factor could be responsible for the *Hsp* gene induction observed in the microarray experiments, suggesting a similar mechanism of gene regulation to the one we previously demonstrated in cortical neurons (Pignataro et al. 2007).

**Figure 4:**
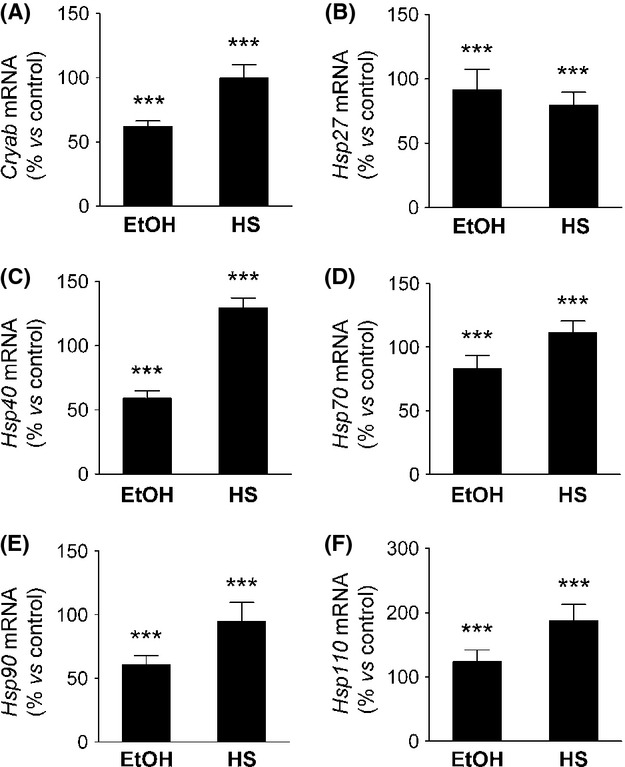
Ethanol activates the transcription of heat shock protein (*Hsp*) genes in primary astrocyte culture. (A–F) Increase in *Cryab*, *Hsp27*, *Hsp40*, *Hsp70*, *Hsp90*, and *Hsp110* mRNA after treatment for 1 h with 60 mmol/L ethanol (EtOH) or heat (HS), as measured by Q-PCR. The data were normalized to *Actb* mRNA and compared with control samples by one-way ANOVA with Dunnett's multiple comparison post hoc test, *n* ≥ 8. All data are the mean ± SEM (significantly different at the level of ***P < 0.001).

To confirm that ethanol stimulates HSF1 transcriptional activity in astrocytes, we investigated whether ethanol induced HSF1-dependent transcription of the main members of each *Hsp* gene class. As heat shock strongly stimulates *Hsp* gene transcription, we used this treatment as a positive control (Tonkiss and Calderwood 2005). Exposure of the primary cultures to 60 mmol/L ethanol for 1 h, or to heat shock (42°C, 1 h), rapidly increased the mRNA levels of *Cryab*, *Hsp27*, *Hsp40*, *Hsp70*, *Hsp90*, and *Hsp110* (Fig. 4A–F) as measured using Q-PCR. The results of ethanol exposure on the induction of the *Hsp* genes did not differ significantly when gene expression was normalized with the expression of *Actb* or *Rn18S* cDNA (Fig. S2). All subsequent Q-PCR experiments were performed using *Actb* as the housekeeping gene to standardize for internal differences in RNA content in the samples.

**Figure 5:**
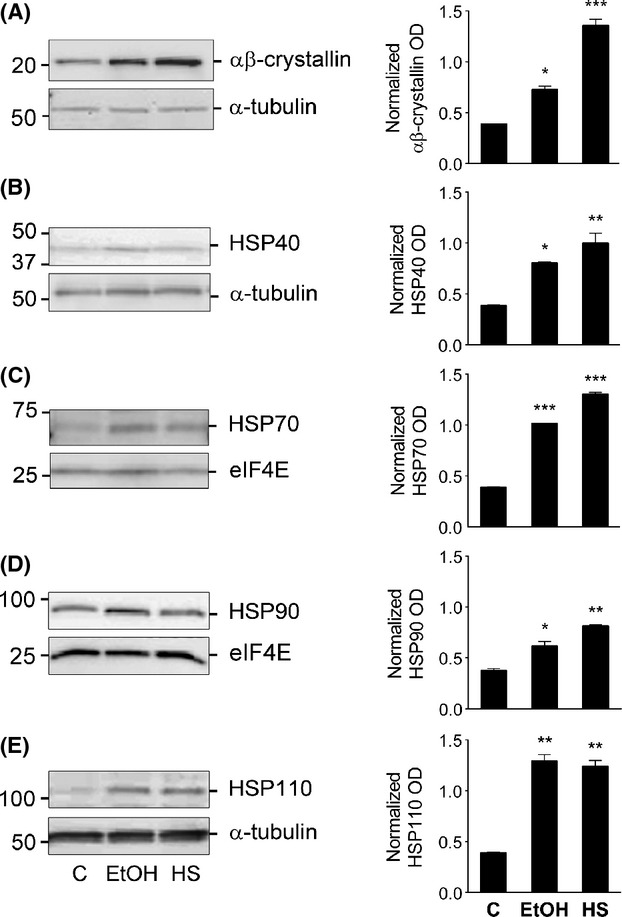
Ethanol induces the expression of heat shock proteins (HSPs) in primary astrocyte culture. (A–E) Increase in αβ-crystallin, HSP40, HSP70, HSP90, and HSP110 protein levels after treatment for 2 h with 60 mmol/L ethanol (EtOH) or 42°C heat shock (HS) in primary astrocyte culture. Representative immunoblots are shown, with the proteins eIF4E or α-tubulin used as internal standards. The bar graphs to the right of the immunoblots represent the quantification of immunoreactive bands intensities normalized to the internal standard, expressed in arbitrary optical density (OD) units. The data are the mean ± SEM of normalized relative OD values analyzed by one-way ANOVA with Dunnett's multiple comparison post hoc test, *n* ≥ 3 (significantly differently at the level of *P < 0.05, **P < 0.01, ***P < 0.001).

Immunoblot analysis of the HSPs confirmed that ethanol and heat shock increase the protein expression levels of αβ-crystallin, HSP40, HSP70, HSP90, and HSP110 in astroctyes (Fig. 5A–E). Interestingly, despite using several different commercial antibodies, we could not detect an immunoreactive band corresponding to HSP27, suggesting that this transcript may not be efficiently translated in astrocyte culture.

### The activation of HSF1 induces a subset of ARGs identified by microarray analysis

**Figure 6:**
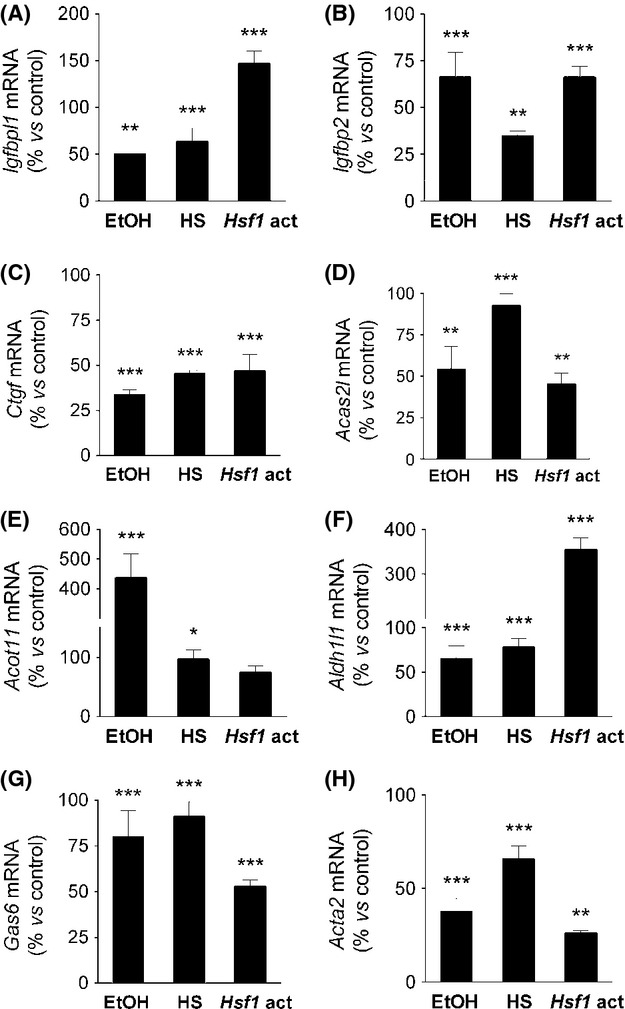
Induction of ethanol- and heat shock-responsive genes by activated heat shock factor 1 (HSF1). (A–H) Increase in *Igfbpl1*, *Igfbp2*, *Ctgf*, *Acas21*, *Acot11*, *Aldh1l1*, *Gas6*, and *Acta2* mRNA after treatment for 1 h with 60 mmol/L ethanol (EtOH) or 42°C heat stress (HS), or transfection with a constitutively active HSF1 construct (*Hsf1-act*) as measured by Q-PCR. The data were normalized to *Actb* mRNA and compared with control samples by one-way ANOVA with Dunnett's multiple comparison post hoc test, *n* ≥ 6. All data are the mean ± SEM (significantly different at the level of *P < 0.01, **P < 0.005, ***P < 0.001).

We next used Q-PCR to assess ethanol-induced changes in the expression of a relevant gene from each of the main gene classes identified in the microarray analyses. Primary astrocyte culture exposed to alcohol and heat stress showed increased expression of all of the selected genes (*Igfbpl1*, *Igfbp2*, *Ctgf*, *Acas21*, *Acot11*, *Aldh1l1*, *Gas6*, and *Acta2*), confirming the microarray results and validating our selection criteria of these genes as ARGs that are likely to be regulated by the transcription factor HSF1 (Fig. 6A–H).

**Figure 7:**
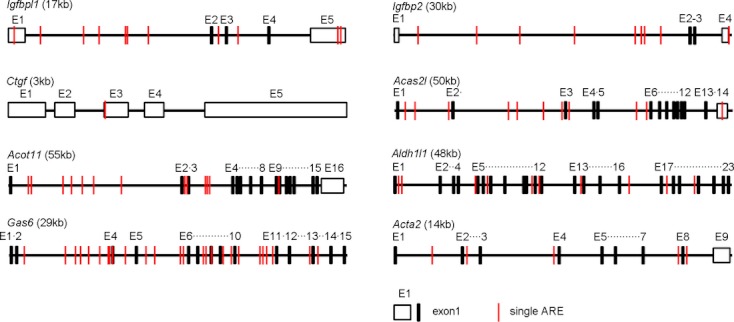
Schematic representation of the location and number of alcohol response elements (ARE) in some of the genes sensitive to both ethanol and heat stress treatments in cortical astrocytes. Note the presence of several ARE either in the proximal 5′ region or downstream in the intron/exon region. The relative position of the introns, exons, and ARE has been conserved in the illustration.

In order to verify that HSF1 transcriptional activity induces the expression of some of the ARGs identified by the microarray experiments, we transfected astrocytes with a constitutively transcriptionally active *Hsf1* construct (*Hsf1-act*) that is capable of inducing the expression of *Hsp* genes in the absence of stress (Acquaah-Mensah et al. 2001). The protocol used for these experiments resulted in a high rate of transfection efficiency of the primary astrocyte culture, evidenced by the significant expression of the *Hsf1* construct (Fig. S3). In astrocytes, transfection of this construct induced the expression of *Igfbpl1*, *Igfbp2*, *Ctgf*, *Acas21*, *Acot11*, *Aldh1l1*, *Gas6*, and *Acta2* genes, mimicking the effects of both ethanol and heat stress (Fig. 6A–H). We have previously identified the neuron-specific gene *Gabra4* as an ethanol- and heat stress-sensitive gene. *Gabra4* gene induction is mediated by the binding of transcriptionally activated HSF1 to a specific sequence within the second intron, which we termed the ARE (Pignataro et al. 2007). The alcohol response element (ARE) is an 11-bp *cis*-regulatory element (tCTGcGTCtCt, uppercase letters indicate absolute conservation) that was first identified in a subset of ARGs in *Caenorhabditis elegans* (Kwon et al. 2004). This element forms a consensus binding site for HSF1, though its sequence is distinct from the classical heat shock element (HSE; Pignataro et al. 2007). Sequence analysis of the genes induced by the *Hsf1* transcriptionally active construct reveal that all of them (*Igfbpl1*, *Igfbp2*, *Ctgf*, *Acas21*, *Acot11*, *Aldh1l1*, *Gas6*, and *Acta2*) contain one or more potential ARE sequence, located either in the proximal 5′-upstream region or downstream in an intronic region, as previously noted in *Gabra4* gene (Fig. 7; Pignataro et al. 2007).

## Discussion

In this study, we performed microarray analyses to determine the effects of acute ethanol exposure on gene expression in primary cultures of mouse cortical astrocytes. We found that ethanol induced 1080 genes and downregulated 1067 genes (Fig. 1 and Table S1). To our knowledge, this is the first study to investigate the genomic adaptative response triggered by alcohol in a relatively pure astrocyte preparation. Previous work performed using tissue from the frontal cortex of human alcoholics has identified a number of glial genes that were differentially regulated (Lewohl et al. 2000; Mayfield et al. 2002; Flatscher-Bader et al. 2005), with 79 astrocyte-specific genes upregulated in the cortex of cirrhotic alcoholics (Liu et al. 2007). These original findings provided the driving force for our present study and when they are considered together with our results indicate that astrocytes are active participants in the genomic response of the brain to ethanol. Our microarray screen shows that a brief exposure of cortical astrocytes to ethanol increased the expression of a large number of genes. These ARGs fall into the class of glial-specific immune response genes, as well as genes involved in the regulation of transcription, cell proliferation and differentiation, and genes of the cytoskeleton and extracellular matrix. Genes involved in metabolism were also upregulated by alcohol exposure, including genes associated with oxidoreductase activity, insulin-like growth factor signaling, acetyl-CoA and lipid metabolism. In contrast, a similar analysis in ethanol-treated primary cortical neurons revealed genes involved in synaptic transmission, calcium sensor proteins involved in vesicle docking to the plasma membrane, synapse formation and plasticity, microtubule assembly and trafficking protein genes (Pignataro et al. 2007). Therefore, the different classes of genes induced by ethanol in astrocytes and neurons appear to be related to the physiological function of each cell type.

### HSF1 is involved in ethanol regulation of astrocyte gene expression

Previously, we identified a set of neuronal ARGs induced by the activation of HSF1 and its subsequent binding to the ARE (Pignataro et al. 2007). To determine whether a set of astrocytic ARGs is regulated in a similar manner, we first investigated the ability of ethanol to activate HSF1 in these cells. Our data show that acute exposure of astrocytes to ethanol promotes the translocation of HSF1 into the nucleus, a prerequisite for the activation of this transcription factor. As it is known that activated HSF1 induces the expression of *Hsp* genes (Morimoto et al. 1998), we tested whether acute ethanol could stimulate the expression of these genes in astrocytes, and found that ethanol increases HSPs mRNA and protein levels. This is particularly interesting as increased expression of *Hsp* genes has been associated with improved cell survival in stress conditions, with HSPs preventing the misfolding and aggregation of proteins that result in cell death (Calabrese et al. 2001; Takuma et al. 2002; Voloboueva et al. 2008; Shin et al. 2009; Arawaka et al. 2010; Kong et al. 2011).

The exact mechanism ethanol employs to activate HSF1 is still controversial. Classically, elevated temperature has been associated with the activation of HSF1 and the heat shock cascade. However, other biochemical events activate HSF1 at normal physiological temperature and there is a consensus within the field that conditions that alter normal protein conformation (temperature, calcium, urea, pH) can also induce HSF1-DNA binding (Mosser et al. 1990). As recent studies have observed that acute ethanol can trigger the release of calcium from internal stores (Kelm et al. 2007, 2008, 2010), we speculate that ethanol may increase free intracellular calcium concentrations to alter protein conformation and activate HSF1 and the heat shock cascade.

To identify candidate ARGs regulated by HSF1 transcriptional activity in our microarray analyses, we selected genes that responded to both ethanol and heat shock treatments. We confirmed the microarray results of some of these physiologically relevant genes from each class of biological function by analyzing their expression in astrocytes exposed to alcohol and heat shock. All the genes tested (*Igfbpl1*, *Igfbp2*, *Ctgf*, *Acas21*, *Acot11*, *Aldh1l1*, *Gas6*, and *Acta2*) showed induction by ethanol, validating them as ARGs and corroborating the selection criteria used to identify the genes from the microarray screens. Furthermore, overexpression of a constitutively transcriptionally active HSF1 in astrocytes induced these ARGs in the absence of alcohol. Finally, sequence analysis of these ARGs identified the presence of one or more candidate ARE sequences in the proximal 5′-upstream region or downstream in the intron/exons region (Fig. 7). Taken together, these data provide strong evidence that, as in neurons, a subset of astrocyte ARGs are regulated by the transcriptional activity of HSF1.

### Effects of ethanol on astrocytes and CNS homeostasis

Astrocytes play an important role maintaining homeostasis and mediating neuroprotection in the CNS. They supply neurons with a variety of metabolic substrates (Vernadakis 1988; Kirchhoff et al. 2001; Wang and Bordey 2008) and protect them against oxidative stress (Aschner and Kimelberg 1991; Kirchhoff et al. 2001; Gonzalez and Salido 2009). It is perhaps not surprising, therefore, that many of the astrocytic genes induced by ethanol in our study are involved in metabolic functions like acetyl-CoA metabolism, nucleotide metabolism, and oxidoreductase activity (Table S1).

Ethanol intake leads to the formation of ROS in the CNS, which can then alter the redox state of astrocytes (Russo et al. 2001; Gonthier et al. 2004; Schiaffonati 2005). As the CNS does not express alcohol dehydrogenase, ethanol is metabolized within astrocytes by catalase or cytochome P450, a part of the microsomal ethanol oxidizing system that generates ROS. In this process, ethanol is initially metabolized to acetaldehyde, which is then converted to acetate and acetyl-CoA. In addition to ROS generation (Montoliu et al. 1995; Russo et al. 2001; Muscoli et al. 2002; Gonzalez et al. 2007), ethanol also interferes with the normal absorption, biosynthesis, metabolism, and function of antioxidants, making astrocytes more sensitive to oxidative damage (Montoliu et al. 1995; Gonthier et al. 2004; Gonzalez et al. 2007).

We found that ethanol exposure increased the expression of genes involved in oxidoreductase activity and the generation of antioxidant enzymes, such as *Lox*, *Loxl1*, *Tst*, *Plin3*, *Cyp1b1*, *Gstt3*, *Aldh1l1*, and *Cp* (Table S1). Lysyl oxidase and lysyl oxidase-like 1 genes (*Lox and Loxl1*) encode copper-dependent lysine oxidases that allow the cross-linking of extracellular matrix proteins (Lucero and Kagan 2006; Rodriguez et al. 2008). These enzymes are also well known to be induced by alcohol in the liver, and contribute to the fibrosis seen in chronic alcoholics (Shiota et al. 1987). Most of the other genes upregulated in this category act to enhance antioxidants. For instance, cyanide sulfurtransferase or rhodanase (*Tst*) forms antioxidant sulfane sulfur compounds (Iciek and Wlodek 2001) and gluthatione-*S*-transferase theta 3 (*Gstt3*) synthesizes the antioxidant gluthatione (Knight et al. 2008). Additionally, *Plin3* associates with the mitochondria during oxidative stress to protect cells from hydrogen peroxide–induced cell death (Hocsak et al. 2010), while aldehyde dehydrogenase (*Aldh1l1*) detoxifies aldehyde substrates from astrocytes via NAD(P)^+^-dependent oxidation (Yang et al. 2011). Finally, ceruloplasmin (*Cp*) neutralizes the harmful effect of excess free copper and iron and stimulates the release of ROS (Negru et al. 1999). Overall, these findings suggest that alcohol exposure induces metabolic and oxidoreductase gene expression in astrocytes to protect these cells and the entire CNS from ethanol-induced oxidative damage.

### Apoptosis

The hyperoxidative state produced by ethanol in astrocytes can trigger apoptosis in some functionally impaired cells (Russo et al. 2001; Schiaffonati 2005; Bell et al. 2009; Lu et al. 2010), and accordingly, we found that ethanol induced several apoptosis regulation genes in our microarray study (*Col18al*, *Rtn1*, *Pea15*, *Idb4*, and *Insl6*). *Col18al* encodes procollagen XVIII (Kague et al. 2010), and the C-terminal fragment of this protein produces endostatin, a potent promoter of apoptosis and an angiogenesis inhibitor (Hanai et al. 2002; Heljasvaara et al. 2005). *Rtn1* encodes for the chaperone protein reticulon 1, which induces apoptosis by sensing CNS endoplasmic reticulum stress (Di Sano et al. 2007). Similarly, the gene product known as phosphoprotein enriched in astrocytes 15 kDa/phopsphoprotein enriched in diabetes (PEA-15/PED) is a death effector domain-containing protein that modulates TNF-α-induced apoptosis in astrocytes (Bock et al. 2010). The inhibitor of DNA-binding *(Idb4)* gene regulates astrocytic apoptosis via cAMP-dependent signaling (Andres-Barquin et al. 1999), while a deficiency in insulin like 6/relaxin-like gene (*Insl6/RIF1*) in mice also enhances apoptosis (Brailoiu et al. 2005; Burnicka-Turek et al. 2009). The activation of this set of genes is consistent with the hypothesis that ethanol may induce apoptosis in a subset of astrocytes in response to oxidative damage.

### Insulin-like growth factor signaling

Insulin-like growth factor (ILGF) signaling, which regulates cellular proliferation and survival, is strongly associated with the liver damage produced by ethanol consumption (Adamo et al. 1992; Park et al. 2004). In the brain, ethanol is known to increase insulin-like growth factor binding proteins (IGFBP) that mediate the effects of ILGF (Kumar et al. 2002; Dalcik et al. 2009a). In our microarray experiments, we observed the induction of *Igfbp2*, a gene that has also been shown to regulate the proliferation, invasion, and angiogenesis of glioblastomas (Fukushima and Kataoka 2007). We also detected increased expression of *Igfbpl1*, another gene associated with cancer cell proliferation (Smith et al. 2007). Several other genes related to this superfamily of growth factors were induced in our experiments, including connective tissue growth factor (*Ctgf*), which codes for a member of the IGFBP superfamily that modulates the mitotic actions of insulin-like growth factors in astrocytes (Kim et al. 1997; Schwab et al. 2000, 2001). As the IGFBP superfamily mediates ILGF signaling activity, it is possible that ethanol's effects on its expression levels may be linked to the CNS damage caused by chronic alcohol consumption.

### Genes involved in inflammation and immunity

There is increasing consensus within the field that inflammation plays a significant role in the neurodegeneration seen in the brains of chronic alcoholics (Valles et al. 2004; Pascual et al. 2007). Astrocytes, as well as microglia, have been proposed as cellular participants in this ethanol-induced neurodegeneration (Tacconi 1998; Norenberg 2005; Crews et al. 2006; Farina et al. 2007), and chronic ethanol treatment has been shown to activate IL-1β in astrocytes, both in vivo and in vitro (Blanco et al. 2004, 2005; Valles et al. 2004; Guasch et al. 2007). It is thought that that this immune response may be triggered in part by the appearance of metabolic adducts formed from the reaction of the ethanol metabolite acetaldehyde with proteins, nucleic acids, and phospholipids (Deitrich et al. 2006; Zimatkin et al. 2006). These adducts are recognized as ‘foreign’ molecules within the body and stimulate an immune response. In support of this hypothesis, researchers have identified antibodies against acetaldehyde-containing adducts in the liver (Clot et al. 1997; Niemela 2001; Worrall and Thiele 2001; Tuma 2002), suggesting that a similar process could activate an immune response in the CNS.

In our microarray experiments, we found that acute ethanol rapidly induces several genes that regulate the cellular immune response and participate in the production of inflammatory soluble intermediates, including *Pea15*, *Rsg16*, *Cd97*, *Entpd2*, *Gas6*, and *Fdz5*. Alcohol regulation of the cellular immune response is mediated by PEA-15/PED, which decreases T-cell proliferation (Pastorino et al. 2010) and protects astrocytes from TNF-α-triggered apoptosis (Sharif et al. 2003). *Rsg16* (regulator of G-protein signaling 16) is a GTPase activating protein that regulates chemokine-induced T lymphocytes (Lippert et al. 2003). Finally, *Cd97*, a G-protein coupled receptor and part of the epidermal growth factor receptor (EGFR) class (Hamann et al. 2000), mediates granulocyte and T-cell stimulation (van Pel et al. 2008; Kop et al. 2009).

Alcohol also upregulates a set of genes that control the humoral immune response, including ectonucleoside triphosphate diphosphohydrolase 2 (*Entpd2*), a brain ectonucleotidase that modulates inflammation by controlling the levels of AMP (Wink et al. 2006). Similarly, growth arrest–specific gene 6 (*Gas6*) inhibits the production of TNF-α, IL-1β, IL-6, and iNOS in LPS-stimulated macrophages (Grommes et al. 2008; Alciato et al. 2010). Finally, the receptor Frizzled-5 (*Fdz5*) regulates the IL-12 response via Toll-like receptor signaling and NF-κB activation (Blumenthal et al. 2006). The induction of all these genes is consistent with the notion that astrocytes play a role in mounting a complex immune response after the brain's exposure to alcohol and its metabolites.

### Acetyl-CoA and lipid metabolism

Ethanol can be metabolized by a variety of enzymes, but irrespective of the enzymatic route, the first product is always acetaldehyde, a highly unstable metabolite that quickly forms free radicals. Aldehyde dehydrogenase family 2 rapidly converts acetaldehyde to acetate and NADH, and acetate is then converted into acetyl-CoA by acetyl-CoA synthase (Tuma and Casey 2003; Deitrich et al. 2006). Consequently, it was not a surprise to find that ethanol-treated astrocytes increased the gene expression of acetyl-CoA synthase 2 (*AceCS2 or Acas2l*), the enzyme involved in the trafficking of acetate to and from the mitochondria in the form of acetyl-CoA (Carman et al. 2008). Another set of ethanol-induced genes were acyl-CoA thioesterases (*Acot11* and *Acot1*), which participate in acetate metabolism by hydrolyzing acyl-CoA esters to produce the acetate acceptor CoA (Kirkby et al. 2010). Another ethanol-induced gene encodes the enzyme nucleoside diphosphate-linked moiety X motif 7 (*Nudt7*), which eliminates oxidized CoA from peroxisomes and regulates the cellular levels of CoA and acetyl-CoA (Gasmi and McLennan 2001).

The acetyl-CoA and NADH generated as a consequence of ethanol metabolism can enter the citric acid cycle and then produce ATP via the mitochrondrial electron transport systems. Alternatively, this excess acetyl-CoA can be diverted for cholesterol and lipid synthesis (Zakhari 2006), likely leading to the “fatty liver” observed in alcoholic steatohepatitis (Lieber 2004; Stickel and Seitz 2010). Lipoprotein lipase (*Lpl*) is another important astrocyte ARG that is associated with the increased lipoproteins detected in ethanol drinking mice (Mudrakova and Kovar 2007). The enzymatic activity of this gene as a lipase or acyltransferase enables the accumulation of lipids in conditions of excess calorie intake (Nikonova et al. 2008). Finally, sphingomyelinase-like phosphodiesterase 3a *(Smpdl3a or Asml3a*) regulates the content of sphingomyelin in the plasma membrane and the composition of lipid rafts (Gupta et al. 2010). The upregulation of these acetyl-CoA and lipid metabolism genes in astrocytes exposed to ethanol indicates the crucial role that these cells play in the global CNS response to alcohol.

## Summary and Conclusions

The data presented here indicate that alcohol produces rapid and significant changes in the gene expression patterns of astrocytes. The presence of ethanol alters the redox state of the cells, triggering an increase in the expression of genes related to oxidoreductases, antioxidants, stress, and apoptosis. We also observed the regulation of genes that control the immune response, as well as those involved in acetyl-CoA and lipid metabolism. The data presented here suggest that a significant number of the astrocyte ARGs we identified are regulated by HSF1, perhaps via the ARE. Although we have confirmed several genes within this group, we cannot rule out the existence of a variety of other gene regulatory mechanisms that govern alcohol sensitivity.

Overall, the astrocyte genomic adaptation to ethanol resembles the response seen in the livers of rodents and cultured hepatocytes exposed to ethanol. Microarray studies reveal that ethanol produces oxidative stress and toxicity in cultured hepatocytes, inducing lipid and oxidative stress metabolism genes (Ciuclan et al. 2010). Induction of enzymes involved in oxidative stress was also noted in ethanol-treated mice, with increased gene expression related to lipid metabolism (Bardag-Gorce et al. 2009). Other studies performed on rats exposed to ethanol showed the induction of gene classes in the liver similar to those reported for astrocytes in this study, including glutathione metabolism, apoptosis, cytokine and cytokine receptor, carbohydrate and protein metabolism, and cell structure and cytoskeleton (Bachoo et al. 2004; Deaciuc et al. 2004; Park et al. 2008). The striking similarity of gene categories induced by ethanol in astrocytes and in hepatocytes suggests that alcohol may interact with similar signaling and regulatory mechanisms to regulate gene expression in the brain and the liver. Future efforts will be directed toward extending these studies to longer term alcohol exposures and models of withdrawal to better understand the consequences of alcohol exposure on astrocytes and within the CNS as a whole.

Astrocytes play a crucial role in the CNS, supporting normal neuronal activity by maintaining CNS homeostasis and controlling the concentrations of neurotransmitters and ions in the extracellular space (Vernadakis 1988; Wang and Bordey 2008; Belanger and Magistretti 2009). Ethanol regulation of the heat shock cascade and gene expression in astrocytes, therefore, may have profound implications for neuronal physiology. While there has been no work directly addressing this issue, several studies have shown that HSPs are involved in protecting the brain from a variety of insults, including ischemia and neurodegeneration (Yenari 2002). In particular, it was found that overexpression of HSP72 in astrocytes prior to ischemia prevented astrocytic glutamate transporter dysfunction and subsequent neuronal death in the CA1 region of the hippocampus (Xu et al. 2010). These findings suggest that ethanol activation of the heat shock cascade and induction of the *Hsp* genes in astrocytes may actually protect nearby neurons from any deleterious effects of alcohol exposure, as well as from future insults. Future studies will investigate these secondary effects of alcohol on neurons in order to identify changes in astrocytic gene expression and pathways that may be associated with the neuroprotective effects of alcohol.

## Supporting Information

**Figure S1.** Primary cell cultures from embryonic mouse cortex result in almost pure astrocyte populations.

**Figure S2.** Normalization with two different housekeeping genes does not affect ethanol-induced expression of Hsp70 in primary astrocyte culture.

**Figure S3.** Efficiency of transfection of DNA constructs in primary culture of mouse astrocytes.

**Table S1.** Genes significantly activated by acute ethanol (E, 60 mmol/L, 1 h) and heat shock (HS, 42°C, 1 h) in primary cultures of astrocytes.
