# Supplementary material for: Brief alcohol exposure alters transcription in astrocytes via the heat shock pathway
Source: Brain Behav. 2013 Feb 6;3(2):114–33. doi: 10.1002/brb3.125 (PMC3607153; doi:10.1002/brb3.125)
Supplement: Supplementary file 1 [file brb30003-0114-SD1.docx]

**Supporting Information**


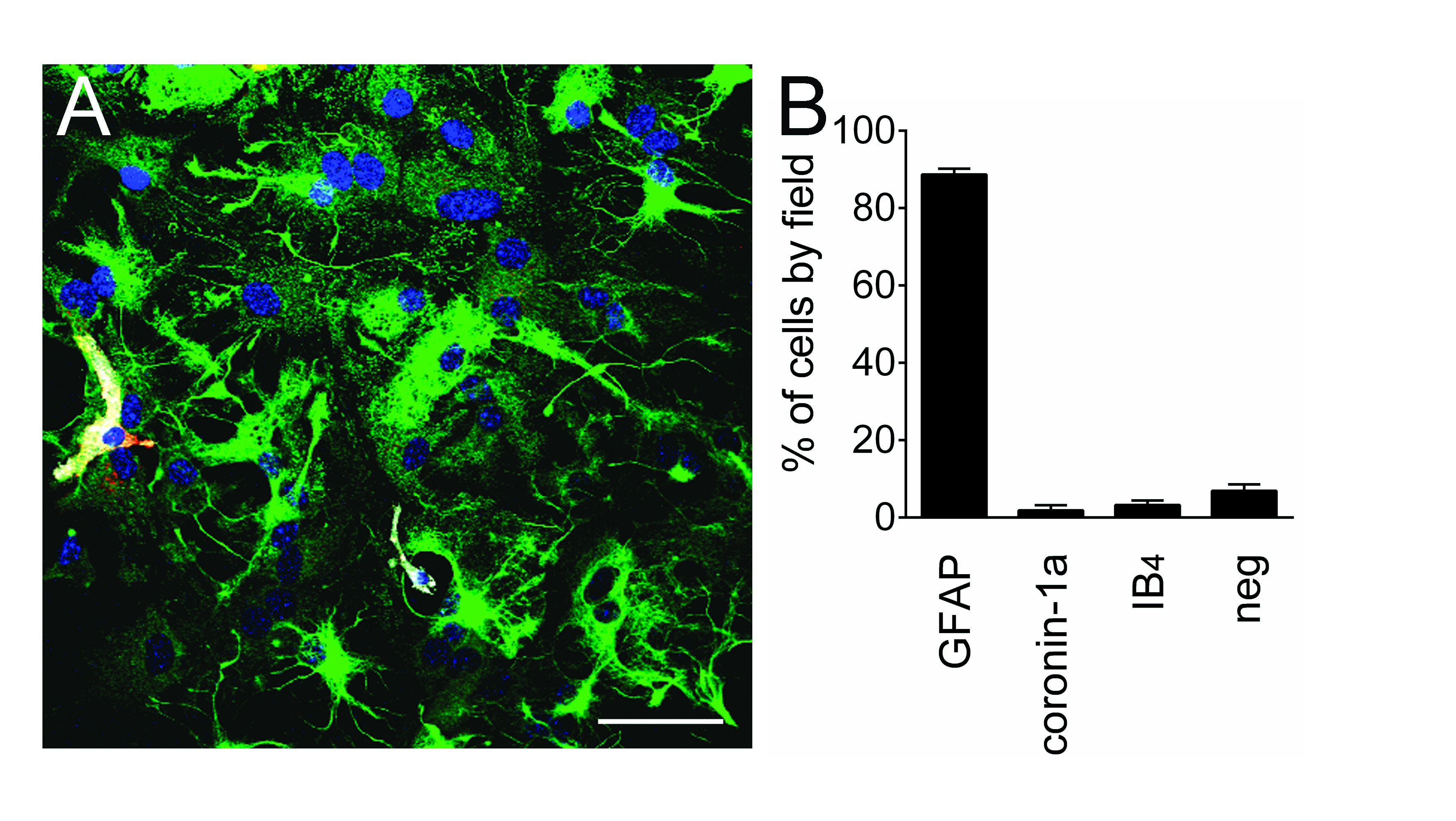


**Supplemental Figure 1**. Primary cell cultures from embryonic mouse cortex result in almost pure astrocyte populations. ***A***, Immunocytochemistry of cell cultures stained with the mature astrocyte marker glial fibrillary acidic protein (GFAP, green), the microglial markers coronin-1a (white) and isolectin-B_4_ (IB_4_, red), and the nuclear staining DAPI (blue) reveals that most of the cells are positive for the mature astrocytic and only a few are positive for the microglial markers. The bar represents 50 μm. ***B***, Quantification of the number of cells positive for the different markers. The data are the mean ± s.e.m. (n = 12 random fields). A small percentage of cells did not display immunoreactivity to any of the glial markers used in the experiment (neg).


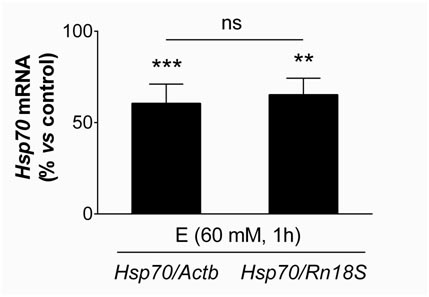


**Supplemental Figure 2**. Normalization with two different housekeeping genes does not affect ethanol induced expression of *Hsp70* in primary astrocyte culture. The expression level of *Hsp70* determined by real-time quantitative RT-PCR was normalized to *Actb* and *Rn18S* (rRNA 18S gene) (QuantumRNA Internal Standards, Ambion) in the same sample, and the results were expressed as percentage of increase above the control (untreated cells or cells treated with vehicle). There was no significant difference between the data normalized with the two housekeeping genes. The data are the mean ± s.e.m and was compared to control samples by one-way ANOVA with Dunnett’s Multiple Comparison *post hoc* test, n ≥ 3 (significantly different *vs.* control at the level of **P<0.005, ***P < 0.001; ns, non-significant different between *Actb* and *Rn18S*).


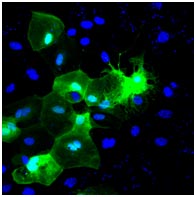

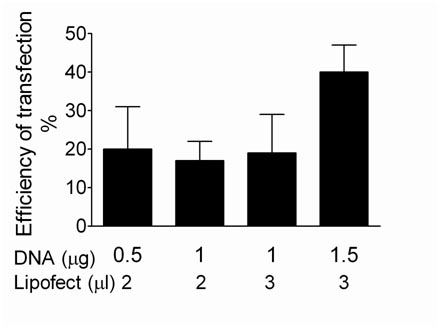


**Supplemental Figure 3**. Efficiency of transfection of DNA constructs in primary culture of mouse astrocytes. Quantification of the number of green fluorescent protein (GFP) positive cells after transfection with different DNA concentrations of a pcDNA3.1^+^ vector containing the fluorescent protein and various amounts of Lipofectamine Plus reagent (Invitrogen). Representative image of the GFP expressing astrocytes stained with the nuclear dye DAPI (blue).
