# Supplementary material for: Brief alcohol exposure alters transcription in astrocytes via the heat shock pathway
Source: Brain Behav. 2013 Feb 6;3(2):114–33. doi: 10.1002/brb3.125 (PMC3607153; doi:10.1002/brb3.125)
Supplement: Supplementary file 2 [file brb30003-0114-SD2.docx]

Supplemental Table 1. Genes significantly activated by acute ethanol (E, 60 mM, 1h) and heat shock (HS, 42°C, 1h) in primary cultures of astrocytes

| Symbol | Acc Number | p-value | CvsE | CvsHS | Definition |
| --- | --- | --- | --- | --- | --- |
| *Fas* | NM_007987.1 | 0.001 | 2.80 | 2.59 | Fas (TNF receptor superfamily member) |
| *Lgals3* | NM_010705.1 | 0.001 | 65.53 | 72.30 | lectin, galactose binding, soluble 3 |
| *Ctgf* | NM_010217.1 | 0.002 | 11.02 | 15.12 | connective tissue growth factor |
| *Crip2* | NM_024223.1 | 0.002 | 5.30 | 4.73 | cysteine rich protein 2 |
| *Pdgfb* | NM_011057.2 | 0.002 | 3.38 | 4.36 | platelet derived growth factor, B polypeptide |
| *Eif2s3y* | NM_012011.1 | 0.002 | 5.92 | 5.58 | eukaryotic translation initiation factor 2, subunit 3, structural gene Y-linked |
| *Lmyc1* | NM_008506.2 | 0.002 | 2.75 | 2.53 | v-myc myelocytomatosis viral oncogene homolog 1, lung carcinoma derived (avian) |
| *Oat* | NM_016978.1 | 0.002 | 2.71 | 2.23 | ornithine aminotransferase |
| *Grb10* | NM_010345 | 0.002 | 5.18 | 6.48 | growth factor receptor bound protein 10 |
| *Igfbpl1* | NM_018741.1 | 0.002 | 21.05 | 19.28 | insulin-like growth factor binding protein-like 1 |
| *Pif1* | NM_172453.1 | 0.003 | 1.21 | -1.15 | PIF1 5'-to-3' DNA helicase homolog (S. cerevisiae) |
| *Idb4* | NM_031166.1 | 0.003 | 3.59 | 4.09 | inhibitor of DNA binding 4 |
| *Igfbp2* | NM_008342.2 | 0.003 | 14.96 | 17.18 | insulin-like growth factor binding protein 2 |
| *Prelp* | NM_054077.2 | 0.003 | 3.22 | 3.03 | proline arginine-rich end leucine-rich repeat |
| *Hey1* | NM_010423.1 | 0.003 | 2.42 | 2.46 | hairy/enhancer-of-split related with YRPW motif 1 |
| *Dhrs1* | NM_026819.2 | 0.003 | 2.32 | 1.99 | dehydrogenase/reductase (SDR family) member 1 |
| *Akr1e1* | NM_018859.1 | 0.003 | 2.45 | 2.38 | aldo-keto reductase family 1, member E1 |
| *Gstt3* | NM_133994.2 | 0.003 | 4.83 | 4.14 | glutathione S-transferase, theta 3 |
| *Taf13* | NM_025444 | 0.003 | 2.35 | 2.47 | TAF13 RNA polymerase II, TATA box binding protein |
| *Catnd2* | NM_008729.1 | 0.003 | 1.58 | 1.49 | catenin (cadherin associated protein), delta 2 |
| *Aqp4* | NM_009700 | 0.003 | 12.92 | 10.66 | aquaporin 4 |
| *Col18a1* | NM_009929.2 | 0.003 | 9.98 | 12.86 | procollagen, type XVIII, alpha 1 |
| *Pea15* | NM_008556.1 | 0.003 | 6.32 | 6.23 | phosphoprotein enriched in astrocytes 15 |
| *Rnf44* | NM_134064.1 | 0.003 | 1.36 | 1.35 | ring finger protein 44 |
| *Ceecam1* | NM_207298 | 0.003 | 1.29 | 1.01 | cerebral endothelial cell adhesion molecule |
| *Crygs* | NM_009967.1 | 0.004 | 9.34 | 10.30 | crystallin, gamma S |
| *Prkcd* | NM_011103.1 | 0.004 | 2.26 | 2.04 | protein kinase C, delta |
| *Cltb* | NM_028870.1 | 0.004 | 2.60 | 2.88 | clathrin, light polypeptide (Lcb) |
| *Casp1* | NM_009807.1 | 0.004 | 2.38 | 1.77 | caspase 1 |
| *B3gnt5* | NM_054052.2 | 0.004 | 2.24 | 2.00 | UDP-GlcNAc:betaGal beta-1,3-N-acetylglucosaminyltransferase 5 |
| *Avpr1a* | NM_016847.2 | 0.004 | 1.65 | 1.45 | arginine vasopressin receptor 1A |
| *Cobl* | NM_172496.2 | 0.004 | 1.74 | 2.05 | cordon-bleu |
| *Meis1* | NM_010789.1 | 0.004 | 3.18 | 3.12 | myeloid ecotropic viral integration site 1 |
| *Prnp* | NM_011170.1 | 0.004 | 1.44 | 1.23 | prion protein |
| *Tspo* | NM_009775.2 | 0.004 | 2.15 | 2.02 | translocator protein |
| *Zfp771* | NM_177362.2 | 0.004 | 1.19 | 1.31 | zinc finger protein 771 |
| *Amotl2* | NM_019764 | 0.005 | 1.07 | 2.07 | angiomotin-like 2 |
| *Dlg3* | NM_016747.2 | 0.005 | 2.02 | 2.35 | discs, large homolog 3 (Drosophila) |
| *Dlk1* | NM_010052 | 0.005 | 5.01 | 5.76 | delta-like 1 homolog (Drosophila) |
| *Map3k7ip1* | NM_025609.2 | 0.005 | 1.06 | -1.67 | mitogen-activated protein kinase kinase kinase 7 interacting protein 1 |
| *Adssl1* | NM_007421.1 | 0.005 | 4.21 | 4.28 | adenylosuccinate synthetase like 1 |
| *Cd97* | NM_011925.1 | 0.005 | 3.18 | 3.49 | CD97 antigen |
| *Cyp1b1* | NM_009994 | 0.005 | 4.72 | 5.52 | P450, family 1, subfamily b, polypeptide 1 |
| *Wbscr18* | NM_025362.2 | 0.005 | 1.04 | -1.27 | Williams-Beuren syndrome chromosome region 18 homolog |
| *Efs* | NM_010112.2 | 0.005 | 1.77 | 1.60 | embryonal Fyn-associated substrate |
| *Mbc2* | NM_011843.1 | 0.005 | 2.19 | 2.11 | membrane bound C2 domain containing protein |
| *Sox2* | NM_011443.2 | 0.005 | 1.20 | 1.45 | SRY-box containing gene 2 |
| *Tmem166* | NM_145570.1 | 0.005 | 1.51 | 1.79 | transmembrane protein 166 |
| *Car2* | NM_009801.3 | 0.005 | 3.09 | 2.54 | carbonic anhydrase 2 |
| *Cd59a* | NM_007652.2 | 0.005 | 2.90 | 2.31 | CD59a antigen |
| *Abat* | NM_172961.2 | 0.005 | 1.91 | 1.74 | 4-aminobutyrate aminotransferase |
| *Dhx32* | NM_133941.1 | 0.005 | 2.18 | 1.91 | DEAH (Asp-Glu-Ala-His) box polypeptide 32 |
| *Igsf11* | NM_170599.2 | 0.005 | 2.60 | 1.89 | immunoglobulin superfamily, member 11 |
| *Rala* | NM_019491.4 | 0.005 | 1.67 | 2.00 | v-ral simian leukemia viral oncogene homolog A |
| *Slc25a45* | NM_134154.1 | 0.005 | 2.10 | 1.75 | solute carrier family 25, member 45 |
| *Tmem47* | NM_138751.1 | 0.005 | 2.80 | 2.19 | transmembrane protein 47 |
| *Gng7* | NM_010319.3 | 0.005 | 2.69 | 2.11 | guanine nucleotide binding protein (G protein), gamma 7 subunit |
| *Cmtm7* | NM_133978.1 | 0.005 | 5.01 | 5.03 | CKLF-like MARVEL transmembrane domain containing 7 |
| *Htra1* | NM_019564.1 | 0.005 | 1.72 | 1.58 | HtrA serine peptidase 1 |
| *Tmco4* | NM_029857.2 | 0.005 | 3.65 | 2.73 | transmembrane and coiled-coil domains 4 |
| *M6prbp1* | NM_025836.1 | 0.005 | 5.20 | 4.68 | mannose-6-phosphate receptor binding protein 1 |
| *Ilk* | NM_010562.1 | 0.005 | 1.67 | 1.71 | integrin linked kinase |
| *Rnf141* | NM_025999.1 | 0.006 | 1.56 | 1.46 | ring finger protein 141 |
| *Mmp14* | NM_008608.2 | 0.006 | 3.21 | 3.16 | matrix metallopeptidase 14 (membrane-inserted) |
| *Scube3* | NM_001004366.1 | 0.006 | 3.03 | 2.76 | signal peptide, CUB domain, EGF-like 3 |
| *Erlin2* | NM_153592.1 | 0.006 | 1.32 | 1.18 | ER lipid raft associated 2 |
| *Trim47* | NM_172570.2 | 0.006 | 1.89 | 2.23 | tripartite motif protein 47 |
| *Prr7* | NM_001030296.2 | 0.006 | 3.05 | 2.98 | proline rich 7 (synaptic) |
| *Tmem108* | NM_178638.2 | 0.006 | 2.38 | 1.61 | transmembrane protein 108 |
| *Grm8* | NM_008174 | 0.006 | 1.44 | 1.67 | glutamate receptor, metabotropic 8 |
| *Hpxn* | NM_017371.1 | 0.006 | 1.05 | 1.07 | hemopexin |
| *Nkx2-2* | NM_010919 | 0.006 | 2.84 | 2.95 | NK2 transcription factor related, locus 2 |
| *Fbxw17* | NM_175401.2 | 0.006 | 1.39 | 1.18 | F-box and WD-40 domain protein 17 |
| *Gprin1* | NM_012014.1 | 0.006 | 2.17 | 1.69 | G protein-regulated inducer of neurite outgrowth 1 |
| *Insl6* | NM_013754.1 | 0.006 | 3.05 | 2.33 | insulin-like 6 |
| *Ipmk* | XM_125641.3 | 0.006 | 1.45 | 1.40 | inositol polyphosphate multikinase |
| *Mettl9* | NM_021554.2 | 0.006 | 1.43 | 1.47 | methyltransferase like 9 |
| *Pkia* | NM_008862.2 | 0.006 | 2.97 | 3.14 | protein kinase inhibitor, alpha |
| *Prkacb* | NM_011100.3 | 0.006 | 1.04 | -1.10 | protein kinase, cAMP dependent, catalytic, beta |
| *Syt11* | NM_018804 | 0.006 | 1.89 | 1.76 | synaptotagmin XI |
| *Mlf1* | NM_001039543.1 | 0.007 | 2.89 | 2.58 | myeloid leukemia factor 1 |
| *Kcnab1* | NM_010597.2 | 0.007 | 1.89 | 1.79 | potassium voltage-gated channel, shaker-related subfamily, beta member 1 |
| *Arl4c* | NM_177305.3 | 0.007 | 2.61 | 2.38 | ADP-ribosylation factor-like 4C |
| *E2f6* | NM_033270.1 | 0.007 | 1.41 | 1.79 | E2F transcription factor 6 |
| *Tra1* | NM_011631.1 | 0.007 | 1.13 | 1.40 | heat shock protein 90kDa beta |
| *Fbln1* | NM_010180.1 | 0.007 | 11.22 | 11.17 | fibulin 1 |
| *Mak10* | NM_030153.1 | 0.007 | 1.42 | 1.37 | MAK10 homolog, amino-acid N-acetyltransferase subunit, (S. cerevisiae) |
| *Tmc6* | NM_145439.1 | 0.007 | 1.76 | 1.55 | transmembrane channel-like gene family 6 |
| *Ssx2ip* | NM_138744.2 | 0.007 | 3.06 | 2.63 | synovial sarcoma, X breakpoint 2 interacting protein |
| *Nipa1* | NM_153578.1 | 0.007 | 2.62 | 2.50 | non imprinted in Prader-Willi/Angelman syndrome 1 homolog |
| *Tbc1d7* | NM_025935.1 | 0.007 | 1.85 | 1.63 | TBC1 domain family, member 7 |
| *Zfp277* | NM_178845.1 | 0.007 | 1.51 | 1.30 | zinc finger protein 277 |
| *Akap12* | NM_031185.1 | 0.007 | 3.64 | 4.92 | A kinase (PRKA) anchor protein (gravin) 12 |
| *Tmc7* | NM_172476.2 | 0.007 | 2.70 | 2.66 | transmembrane channel-like gene family 7 |
| *Dcbld1* | NM_025705 | 0.007 | 2.19 | 2.10 | discoidin, CUB and LCCL domain containing 1 |
| *Gas6* | NM_019521.1 | 0.007 | 7.82 | 6.79 | growth arrest specific 6 |
| *Ttyh1* | NM_021324.3 | 0.007 | 4.72 | 3.11 | tweety homolog 1 (Drosophila) |
| *Pkp2* | NM_026163.1 | 0.007 | 1.76 | 1.71 | plakophilin 2 |
| *Pvrl2* | NM_008990.2 | 0.007 | 2.07 | 1.70 | poliovirus receptor-related 2 |
| *Aldh1l1* | NM_009656.1 | 0.007 | 1.46 | 1.49 | aldehyde dehydrogenase 1 family, member L1 |
| *Slc6a15* | NM_175328.1 | 0.007 | 3.57 | 3.46 | solute carrier family 6 (neurotransmitter transporter), member 15 |
| *Eml1* | XM_127139.5 | 0.007 | 2.16 | 2.39 | echinoderm microtubule associated protein like 1 |
| *Nudt7* | NM_024437.1 | 0.007 | 3.20 | 2.60 | nudix (nucleoside diphosphate linked moiety X)-type motif 7 |
| *Sgcb* | NM_011890.2 | 0.007 | 1.80 | 1.69 | sarcoglycan, beta (dystrophin-associated glycoprotein) |
| *Ephb2* | XM_204072.3 | 0.007 | 3.42 | 2.82 | Eph receptor B2 |
| *Lpl* | NM_008509.1 | 0.007 | 4.00 | 3.06 | lipoprotein lipase |
| *Nomo1* | NM_153057.2 | 0.007 | 1.03 | 1.47 | nodal modulator 1 |
| *Usp2* | NM_198091.1 | 0.008 | 2.64 | 1.95 | ubiquitin specific protease 2 |
| *Add3* | NM_013758.2 | 0.008 | 2.40 | 2.45 | adducin 3 (gamma) |
| *Capg* | NM_007599 | 0.008 | 2.83 | 2.58 | capping protein (actin filament), gelsolin-like |
| *Ptx3* | NM_008987.2 | 0.008 | 3.28 | 3.76 | pentraxin related gene |
| *Dkk3* | NM_015814.2 | 0.008 | 1.54 | 1.38 | dickkopf homolog 3 (Xenopus laevis) |
| *Ppic* | NM_008908.1 | 0.008 | 1.93 | 1.94 | peptidylprolyl isomerase C |
| *Csrp1* | NM_007791.2 | 0.008 | 2.00 | 1.95 | cysteine and glycine-rich protein 1 |
| *Sgk3* | NM_177547.2 | 0.008 | 1.53 | 1.38 | serum/glucocorticoid regulated kinase 3 |
| *Ard1* | NM_019870.1 | 0.009 | 1.74 | 1.74 | N-acetyltransferase ARD1 homolog (S. cerevisiae) |
| *Lrrc57* | NM_025657.2 | 0.009 | 1.87 | 1.76 | leucine rich repeat containing 57 |
| *Stard8* | NM_199018.1 | 0.009 | 5.78 | 4.62 | START domain containing 8 |
| *Acta2* | NM_007392 | 0.009 | 3.38 | 3.44 | actin, alpha 2, smooth muscle, aorta |
| *Ppp4r1* | NM_146081.1 | 0.009 | 1.05 | 1.34 | protein phosphatase 4, regulatory subunit 1 |
| *Ddx3y* | NM_012008.1 | 0.009 | 6.35 | 5.59 | DEAD (Asp-Glu-Ala-Asp) box polypetide 3y |
| *Acsbg1* | NM_053178.1 | 0.009 | 2.47 | 2.32 | acyl-CoA synthetase bubblegum family member 1 |
| *Cyb561d1* | NM_001081320.1 | 0.009 | 1.01 | -1.44 | cytochrome b-561 domain containing 1 |
| *Snx14* | NM_172926.1 | 0.009 | 1.55 | 1.36 | sorting nexin 14 |
| *Coxvib2* | NM_183405.1 | 0.009 | 2.35 | 2.14 | cytochrome c oxidase subunit VIb polypeptide 2 |
| *Fblim1* | NM_133754.3 | 0.009 | 2.85 | 3.35 | filamin binding LIM protein 1 |
| *Rbbp9* | NM_015754.2 | 0.009 | 1.49 | 1.25 | retinoblastoma binding protein 9 |
| *Vim* | NM_011701.3 | 0.009 | 2.15 | 2.03 | vimentin |
| *Rtn1* | NM_153457.4 | 0.010 | 6.70 | 5.60 | reticulon 1 |
| *Hspa1a* | NM_010479.2 | 0.010 | 1.11 | 18.31 | heat shock protein 1A |
| *Vgf* | NM_001039385.1 | 0.010 | 2.83 | 3.79 | VGF nerve growth factor inducible |
| *Nedd1* | NM_008682.1 | 0.010 | 1.36 | 1.39 | neural precursor cell expressed, developmentally down-regulated gene 1 |
| *Cri2* | NM_198425.1 | 0.010 | 1.97 | 1.58 | CREBBP/EP300 inhibitory protein 2 |
| *Actn4* | NM_021895.2 | 0.010 | 1.91 | 2.66 | actinin alpha 4 |
| *Gclm* | NM_008129.2 | 0.010 | 1.76 | 2.12 | glutamate-cysteine ligase , modifier subunit |
| *Tmem65* | NM_175212.4 | 0.010 | 1.77 | 1.61 | transmembrane protein 65 |
| *Pdlim4* | NM_019417.1 | 0.010 | 3.93 | 3.20 | PDZ and LIM domain 4 |
| *Adra2c* | NM_007418.2 | 0.010 | 1.25 | 1.35 | adrenergic receptor, alpha 2c |
| *Chmp7* | NM_134078.2 | 0.010 | 1.31 | 1.27 | CHMP family, member 7 |
| *Ei24* | NM_007915.1 | 0.010 | 1.77 | 1.72 | etoposide induced 2.4 mRNA |
| *Tuba6* | XM_147357.1 | 0.010 | 6.96 | 6.39 | tubulin alpha 6 |
| *Hps6* | NM_176785.1 | 0.010 | 2.15 | 1.72 | Hermansky-Pudlak syndrome 6 |
| *Cd109* | NM_153098.2 | 0.011 | 1.78 | 1.99 | CD109 antigen |
| *Gsc* | NM_010351.1 | 0.011 | 2.66 | 2.45 | goosecoid |
| *Kcnk2* | NM_010607.1 | 0.011 | 2.82 | 2.76 | potassium channel, subfamily K, member 2 |
| *Nup160* | NM_021512.1 | 0.011 | 1.11 | 1.37 | nucleoporin 160 |
| *Slc1a2* | NM_001077515.1 | 0.011 | 1.82 | 1.83 | solute carrier family 1 (glial high affinity glutamate transporter), member 2 |
| *Zfp537* | NM_172298.1 | 0.011 | 1.51 | 1.41 | zinc finger protein 537 |
| *Rassf3* | NM_138956 | 0.011 | 1.36 | 1.46 | Ras association (RalGDS/AF-6) domain family member 3 |
| *Hs3st3a1* | NM_178870 | 0.011 | 3.08 | 2.67 | heparan sulfate (glucosamine) 3-O-sulfotransferase 3A1 |
| *Metrn* | NM_133719 | 0.011 | 2.25 | 2.17 | meteorin, glial cell differentiation regulator |
| *Ndn* | NM_010882.2 | 0.011 | 3.36 | 3.17 | necdin |
| *Capn2* | NM_009794.1 | 0.011 | 1.65 | 2.25 | calpain 2 |
| *Centa1* | NM_172723.1 | 0.011 | 1.26 | 1.19 | centaurin, alpha 1 |
| *Med25* | NM_029365.1 | 0.011 | 1.82 | 1.50 | mediator of RNA polymerase II transcription, subunit 25 homolog (yeast) |
| *Mt3* | NM_013603.1 | 0.011 | 2.63 | 1.87 | metallothionein 3 |
| *Cltb* | NM_028870.1 | 0.011 | 2.54 | 2.54 | clathrin, light polypeptide (Lcb) |
| *Dmn* | NM_207663.2 | 0.011 | 2.11 | 1.78 | desmuslin |
| *Fmod* | NM_021355.2 | 0.011 | 1.93 | 2.09 | fibromodulin |
| *Nr2e1* | NM_152229.1 | 0.011 | 2.95 | 3.13 | nuclear receptor subfamily 2, group E, member 1 |
| *Tmem56* | NM_178936.3 | 0.011 | 3.70 | 3.02 | transmembrane protein 56 |
| *Fthfd* | NM_027406.1 | 0.011 | 3.15 | 3.13 | aldehyde dehydrogenase 1 family, member L1 |
| *H2afx* | NM_010436.2 | 0.011 | 2.30 | 1.95 | H2A histone family, member X |
| *Ggta1* | NM_010283.1 | 0.011 | 2.65 | 2.36 | glycoprotein galactosyltransferase alpha 1, 3 |
| *Hbs1l* | NM_001042593.1 | 0.011 | 1.52 | 1.68 | Hbs1-like (S. cerevisiae) |
| *Nup107* | NM_134010.1 | 0.011 | 1.06 | 1.23 | nucleoporin 107 |
| *Ppp2r1a* | NM_016891.2 | 0.011 | 1.60 | 1.49 | protein phosphatase 2 (formerly 2A), regulatory subunit A (PR 65) |
| *Hn1* | NM_008258.1 | 0.012 | 1.29 | 1.50 | hematological and neurological expressed sequence 1 |
| *Igsf9* | NM_033608.2 | 0.012 | 2.78 | 2.11 | immunoglobulin superfamily, member 9 |
| *Lfng* | NM_008494 | 0.012 | 1.24 | 1.26 | LFNG O-fucosylpeptide 3-beta-N-acetylglucosaminyltransferase |
| *Loxl1* | NM_010729 | 0.012 | 4.04 | 4.14 | lysyl oxidase-like 1 |
| *Slc16a6* | NM_134038.2 | 0.012 | 1.50 | 1.21 | solute carrier family 16 (monocarboxylic acid transporters), member 6 |
| *Slc2a3* | NM_011401.2 | 0.012 | 1.90 | 1.77 | solute carrier family 2 (facilitated glucose transporter), member 3 |
| *Slco1c1* | NM_021471.1 | 0.012 | 5.15 | 4.35 | solute carrier organic anion transporter family, member 1c1 |
| *Bmpr1a* | NM_009758.3 | 0.012 | 1.60 | 1.48 | bone morphogenetic protein receptor, type 1A |
| *Ick* | NM_019987.1 | 0.012 | 1.40 | 1.33 | intestinal cell kinase |
| *Leprel2* | NM_013534.1 | 0.012 | 1.63 | 1.58 | leprecan-like 2 |
| *Snrpn* | NM_013670 | 0.012 | 2.90 | 2.76 | small nuclear ribonucleoprotein N |
| *Tubb2b* | NM_023716.1 | 0.012 | 1.61 | 1.38 | tubulin, beta 2b |
| *Smpdl3a* | NM_020561.1 | 0.012 | 5.69 | 4.87 | sphingomyelin phosphodiesterase, acid-like 3A |
| *Dpysl2* | NM_009955.2 | 0.012 | 1.22 | 1.27 | dihydropyrimidinase-like 2 |
| *Fars2* | NM_001039189.1 | 0.012 | 1.44 | 1.28 | phenylalanine-tRNA synthetase 2 (mitochondrial) |
| *Klf2* | NM_008452.1 | 0.012 | 1.69 | 1.73 | Kruppel-like factor 2 (lung) |
| *Capzb* | NM_001037761.1 | 0.012 | 2.39 | 1.97 | capping protein (actin filament) muscle Z-line, beta |
| *Dlx2* | NM_010054.1 | 0.012 | 2.80 | 4.07 | distal-less homeobox 2 |
| *Hist1h3a* | NM_013550.3 | 0.012 | 1.58 | 1.91 | histone cluster 1, H3a |
| *Lhfp* | NM_175386.3 | 0.012 | 1.32 | 1.44 | lipoma HMGIC fusion partner |
| *Mtbp* | NM_134092.2 | 0.012 | 1.29 | 1.30 | Mdm2, transformed 3T3 cell double minute p53 binding protein |
| *St6galnac2* | NM_009180.3 | 0.012 | 2.59 | 2.11 | ST6 (alpha-N-acetyl-neuraminyl-2,3-beta-galactosyl-1, 3)-N-acetylgalactosaminide alpha-2,6-sialyltransferase 2 |
| *Stard10* | NM_019990.1 | 0.012 | 2.16 | 2.29 | START domain containing 10 |
| *Pdgfc* | NM_019971 | 0.012 | 1.27 | 1.25 | platelet-derived growth factor, C polypeptide |
| *Rnpep* | NM_145417.1 | 0.012 | 1.59 | 1.64 | arginyl aminopeptidase (aminopeptidase B) |
| *Pxmp3* | NM_008994.2 | 0.012 | 1.40 | 1.27 | peroxisomal membrane protein 3 |
| *Faah* | NM_010173.2 | 0.012 | 1.11 | 1.09 | fatty acid amide hydrolase |
| *Hist2h2bb* | NM_175666.2 | 0.012 | 1.61 | 1.77 | histone cluster 2, H2bb |
| *Abcb9* | NM_019875 | 0.012 | 1.56 | 1.27 | ATP-binding cassette, sub-family B (MDR/TAP), member 9 |
| *Nhlrc1* | NM_175340.2 | 0.012 | 1.32 | 1.25 | NHL repeat containing 1 |
| *Prep* | NM_011156.2 | 0.012 | 1.46 | 1.81 | prolyl endopeptidase |
| *Ccrn4l* | NM_009834.1 | 0.012 | 3.11 | 3.38 | CCR4 carbon catabolite repression 4-like (S. cerevisiae) |
| *Hist1h4f* | NM_175655.1 | 0.012 | 1.29 | 1.37 | histone cluster 1, H4f |
| *Qars* | NM_133794.1 | 0.012 | 1.48 | 1.33 | glutaminyl-tRNA synthetase |
| *Agpat3* | NM_053014.2 | 0.012 | 1.96 | 2.36 | 1-acylglycerol-3-phosphate O-acyltransferase 3 |
| *Sfrs5* | NM_001079695.1 | 0.012 | 1.03 | -1.69 | splicing factor, arginine/serine-rich 5 (SRp40, HRS) |
| *Rfx4* | NM_001024918.1 | 0.012 | 2.88 | 2.54 | regulatory factor X, 4 (influences HLA class II expression) |
| *Tst* | NM_009437.2 | 0.012 | 5.49 | 4.33 | thiosulfate sulfurtransferase, mitochondrial |
| *Glo1* | NM_025374.2 | 0.012 | 2.14 | 1.93 | glyoxalase 1 |
| *Il17d* | NM_145837.1 | 0.012 | 1.87 | 1.94 | interleukin 17D |
| *Sucnr1* | NM_032400.1 | 0.012 | 2.14 | 1.61 | succinate receptor 1 |
| *Tpm2* | NM_009416.2 | 0.012 | 5.03 | 5.01 | tropomyosin 2, beta |
| *Cdh2* | NM_007664.1 | 0.012 | 2.13 | 3.31 | cadherin 2 |
| *Entpd2* | NM_009849.1 | 0.012 | 8.03 | 6.27 | ectonucleoside triphosphate diphosphohydrolase 2 |
| *Lims2* | NM_144862.1 | 0.012 | 2.14 | 2.23 | LIM and senescent cell antigen like domains 2 |
| *Acaa2* | NM_177470 | 0.013 | 1.11 | -1.09 | acetyl-Coenzyme A acyltransferase 2 (mitochondrial 3-oxoacyl-Coenzyme A thiolase) |
| *Bace1* | NM_011792.3 | 0.013 | 1.47 | 1.40 | beta-site APP cleaving enzyme 1 |
| *Mest* | NM_008590.1 | 0.013 | 4.35 | 4.68 | mesoderm specific transcript |
| *Slc16a9* | NM_025807.1 | 0.013 | 1.96 | 1.45 | solute carrier family 16 (monocarboxylic acid transporters), member 9 |
| *Nlgn3* | NM_172932.1 | 0.013 | 2.14 | 1.99 | neuroligin 3 |
| *Pskh1* | NM_173432.2 | 0.013 | 1.07 | -1.39 | protein serine kinase H1 |
| *Taf9b* | NM_001001176.1 | 0.013 | 2.15 | 1.78 | TAF9B RNA polymerase II, TATA box binding protein (TBP)-associated factor |
| *Gstk1* | NM_029555.2 | 0.013 | 1.95 | 1.49 | glutathione S-transferase kappa 1 |
| *Zfp41* | NM_001044718.1 | 0.013 | 1.12 | -1.31 | zinc finger protein 41 |
| *Reep1* | NM_178608.2 | 0.013 | 1.30 | 1.12 | receptor accessory protein 1 |
| *Anp32a* | NM_009672.2 | 0.013 | 1.98 | 1.84 | acidic (leucine-rich) nuclear phosphoprotein 32 family, member A |
| *Dstn* | NM_019771.1 | 0.013 | 1.85 | 2.15 | destrin |
| *Vamp2* | NM_009497.2 | 0.013 | 1.35 | -1.10 | vesicle-associated membrane protein 2 |
| *Tmem17* | NM_153596.1 | 0.013 | 1.28 | -1.04 | transmembrane protein 17 |
| *Ctnnbl1* | NM_025680.2 | 0.013 | 1.37 | 1.36 | catenin, beta like 1 |
| *Gpr85* | NM_145066 | 0.013 | 1.49 | 1.32 | G protein-coupled receptor 85 |
| *Ankrd38* | NM_172872.2 | 0.013 | 1.60 | 1.67 | ankyrin repeat domain 38 |
| *En2* | NM_010134.1 | 0.013 | 2.33 | 2.08 | engrailed 2 |
| *Fbxo34* | NM_030236.1 | 0.013 | 1.15 | 1.20 | F-box protein 34 |
| *Mdm2* | NM_010786.2 | 0.013 | 1.21 | 1.78 | transformed mouse 3T3 cell double minute 2 |
| *Acas2l* | NM_080575.1 | 0.013 | 4.89 | 4.63 | acyl-CoA synthetase short-chain family member 1 |
| *Vit* | XM_128764.3 | 0.013 | 3.71 | 3.44 | vitrin |
| *Adk* | NM_134079.1 | 0.013 | 1.46 | 1.49 | adenosine kinase |
| *Epb4.9* | NM_013514 | 0.013 | 1.54 | 1.37 | erythrocyte protein band 4.9 |
| *Eftud2* | NM_011431.2 | 0.013 | 1.25 | 1.16 | elongation factor Tu GTP binding domain containing 2 |
| *Dbp* | NM_016974.1 | 0.014 | 2.16 | 1.79 | D site albumin promoter binding protein |
| *Dyrk3* | NM_145508 | 0.014 | 1.32 | 1.14 | dual-specificity tyrosine-(Y)-phosphorylation regulated kinase 3 |
| *Bzrap1* | NM_172449.1 | 0.014 | 4.37 | 4.73 | benzodiazapine receptor associated protein 1 |
| *Fbxo16* | NM_015795.1 | 0.014 | 1.56 | 1.60 | F-box protein 16 |
| *Gadd45g* | NM_011817.1 | 0.014 | 1.35 | 2.93 | growth arrest and DNA-damage-inducible 45 gamma |
| *Ctsh* | NM_007801.1 | 0.014 | 2.44 | 2.47 | cathepsin H |
| *Mt2* | NM_008630.1 | 0.014 | 1.34 | 1.18 | metallothionein 2 |
| *Acot1* | NM_012006.2 | 0.014 | 3.92 | 2.78 | acyl-CoA thioesterase 1 |
| *Fahd2a* | NM_029629.1 | 0.014 | 1.35 | 1.34 | fumarylacetoacetate hydrolase domain containing 2A |
| *Mcc* | NM_001033406.1 | 0.014 | 2.06 | 2.32 | mutated in colorectal cancers |
| *Gjb3* | NM_008126.1 | 0.014 | 2.18 | 2.10 | gap junction membrane channel protein beta 3 |
| *Eps15* | NM_007943 | 0.014 | 1.37 | 1.43 | epidermal growth factor receptor pathway substrate 15 |
| *Col4a5* | NM_007736 | 0.014 | 2.04 | 2.37 | collagen, type IV, alpha 5 |
| *Lmo6* | NM_175097.2 | 0.014 | 1.51 | 1.61 | LIM domain only 6 |
| *Pon2* | NM_183308.1 | 0.014 | 1.41 | 1.22 | paraoxonase 2 |
| *Bcl2l1* | NM_009743.2 | 0.014 | 1.56 | 1.64 | Bcl2-like 1 |
| *Zhx3* | NM_177263 | 0.014 | 1.41 | 1.33 | zinc fingers and homeoboxes 3 |
| *Smad6* | NM_008542 | 0.014 | 2.02 | 2.11 | MAD homolog 6 (Drosophila) |
| *Chst8* | NM_175140.3 | 0.014 | 1.18 | 1.10 | carbohydrate (N-acetylgalactosamine 4-0) sulfotransferase 8 |
| *Zfp27* | NM_011754.1 | 0.014 | 1.16 | 1.34 | zinc finger protein 27 |
| *Mir16* | NM_019580.3 | 0.014 | 1.46 | 1.68 | membrane interacting protein of RGS16 |
| *Snrk* | NM_133741.1 | 0.014 | 1.30 | 1.26 | SNF related kinase |
| *Ddit4* | NM_029083.1 | 0.014 | 1.26 | 1.77 | DNA-damage-inducible transcript 4 |
| *Egln3* | NM_028133.1 | 0.014 | 4.36 | 3.42 | EGL nine homolog 3 (C. elegans) |
| *Fzd3* | NM_021458.1 | 0.014 | 1.77 | 2.17 | frizzled homolog 3 (Drosophila) |
| *Lox* | NM_010728.1 | 0.014 | 6.02 | 8.33 | lysyl oxidase |
| *Cp* | NM_007752.2 | 0.014 | 3.32 | 3.22 | ceruloplasmin |
| *Sept10* | NM_001024910.1 | 0.014 | 1.36 | 1.35 | septin 10 |
| *Usp20* | NM_028846.1 | 0.014 | 1.49 | 1.31 | ubiquitin specific protease 20 |
| *Olig1* | NM_016968.2 | 0.014 | 4.19 | 3.69 | oligodendrocyte transcription factor 1 |
| *Bgn* | NM_007542.3 | 0.014 | 1.85 | 1.75 | biglycan |
| *Gabrb3* | NM_001038701.1 | 0.014 | 2.16 | 2.08 | gamma-aminobutyric acid (GABA-A) receptor, subunit beta 3 |
| *Rgs16* | NM_011267.1 | 0.015 | 5.68 | 10.14 | regulator of G-protein signaling 16 |
| *Golph2* | NM_027307 | 0.015 | 1.75 | 1.58 | golgi membrane protein 1 |
| *Rbm5* | NM_148930.2 | 0.015 | 1.15 | -1.13 | RNA binding motif protein 5 |
| *Samhd1* | NM_018851.2 | 0.015 | 2.39 | 2.96 | SAM domain and HD domain, 1 |
| *Fgf13* | NM_010200.2 | 0.015 | 2.22 | 2.37 | fibroblast growth factor 13 |
| *Aldh5a1* | NM_172532 | 0.015 | 1.64 | 1.57 | aldhehyde dehydrogenase family 5, subfamily A1 |
| *Itga1* | NM_001033228.1 | 0.015 | 1.48 | 1.96 | integrin alpha 1 |
| *Cenpp* | NM_025495.1 | 0.015 | 1.83 | 1.77 | centromere protein P |
| *Lrrc1* | NM_172528.2 | 0.015 | 1.34 | 1.34 | leucine rich repeat containing 1 |
| *Tagln* | NM_011526 | 0.015 | 3.95 | 4.16 | transgelin |
| *Wnt5a* | NM_009524.2 | 0.015 | 1.22 | 1.29 | wingless-related MMTV integration site 5A |
| *Dcamkl2* | NM_027539.3 | 0.015 | 1.78 | 1.61 | doublecortin-like kinase 2 |
| *Rnase4* | NM_021472.1 | 0.015 | 7.05 | 5.81 | ribonuclease, RNase A family 4 |
| *Ak3* | NM_021299.1 | 0.015 | 1.86 | 1.82 | adenylate kinase 3 |
| *Fzr1* | NM_019757.1 | 0.015 | 1.54 | 1.56 | fizzy/cell division cycle 20 related 1 (Drosophila) |
| *Gstm2* | NM_008183.2 | 0.015 | 1.70 | 1.28 | glutathione S-transferase, mu 2 |
| *Slc44a1* | NM_133891.2 | 0.015 | 1.09 | -1.17 | solute carrier family 44, member 1 |
| *Sorbs1* | NM_001034964.1 | 0.015 | 2.65 | 2.12 | sorbin and SH3 domain containing 1 |
| *Timp3* | NM_011595.1 | 0.015 | 1.76 | 1.75 | tissue inhibitor of metalloproteinase 3 |
| *Cntnap2* | XM_358363.1 | 0.015 | 5.76 | 5.21 | contactin associated protein-like 2 |
| *Slc30a1* | NM_009579 | 0.015 | 1.49 | 1.41 | solute carrier family 30 (zinc transporter), member 1 |
| *Cyp7b1* | NM_007825.1 | 0.015 | 2.37 | 2.27 | cytochrome P450, family 7, subfamily b, polypeptide 1 |
| *Efna5* | NM_207654.1 | 0.015 | 1.35 | 1.12 | ephrin A5 |
| *Gpm6a* | NM_153581.2 | 0.015 | 2.61 | 2.46 | glycoprotein m6a |
| *Syt9* | NM_021889.2 | 0.015 | 2.25 | 1.73 | synaptotagmin IX |
| *Triobp* | NM_138579.2 | 0.015 | 1.57 | 1.49 | TRIO and F-actin binding protein |
| *Ascc1* | NM_026937.1 | 0.015 | 1.21 | 1.24 | activating signal cointegrator 1 complex subunit 1 |
| *Mageh1* | NM_023788.3 | 0.015 | 1.15 | 1.01 | melanoma antigen, family H, 1 |
| *Gldc* | NM_138595.1 | 0.015 | 2.14 | 1.93 | glycine decarboxylase |
| *Asb13* | NM_178283.3 | 0.015 | 1.68 | 1.21 | ankyrin repeat and SOCS box-containing protein 13 |
| *Cd81* | NM_133655.1 | 0.015 | 1.25 | 1.28 | CD 81 antigen |
| *Ebpl* | NM_026598.1 | 0.015 | 1.12 | -1.22 | emopamil binding protein-like |
| *Gmpr* | NM_025508.1 | 0.015 | 2.22 | 1.93 | guanosine monophosphate reductase |
| *Rps6ka1* | NM_009097.1 | 0.015 | 2.08 | 1.76 | ribosomal protein S6 kinase polypeptide 1 |
| *Capn5* | NM_007602.2 | 0.015 | 1.48 | 1.15 | calpain 5 |
| *Kif1b* | NM_008441.2 | 0.015 | 1.06 | 1.25 | kinesin family member 1B |
| *Tm4sf8* | NM_019793.2 | 0.015 | 1.83 | 1.58 | tetraspanin 3 |
| *Ddx25* | NM_013932.2 | 0.015 | 2.31 | 1.98 | DEAD (Asp-Glu-Ala-Asp) box polypeptide 25 |
| *Sptlc1* | NM_009269.2 | 0.015 | 1.58 | 1.25 | serine palmitoyltransferase, long chain base subunit 1 |
| *Arhgef19* | NM_172520.1 | 0.015 | 3.53 | 3.00 | Rho guanine nucleotide exchange factor (GEF) 19 |
| *Ddit4l* | NM_030143.2 | 0.015 | 2.99 | 3.53 | DNA-damage-inducible transcript 4-like |
| *Stard7* | NM_139308.1 | 0.015 | 1.21 | 1.35 | START domain containing 7 |
| *Schip1* | NM_013928.2 | 0.016 | 2.12 | 2.23 | schwannomin interacting protein 1 |
| *Ugt1a6a* | NM_145079.2 | 0.016 | 1.12 | -1.74 | UDP glucuronosyltransferase 1 family, polypeptide A6A |
| *Irf2bp1* | NM_178757.3 | 0.016 | 1.33 | 1.35 | interferon regulatory factor 2 binding protein 1 |
| *Tgfb1i1* | NM_009365.2 | 0.016 | 2.78 | 2.64 | transforming growth factor beta 1 induced transcript 1 |
| *Adrbk1* | NM_130863.1 | 0.016 | 1.01 | 1.25 | adrenergic receptor kinase, beta 1 |
| *Ccdc5* | NM_146089.1 | 0.016 | 1.58 | 1.56 | coiled-coil domain containing 5 |
| *Gstm1* | NM_010358.2 | 0.016 | 1.79 | 1.43 | glutathione S-transferase, mu 1 |
| *Prkcdbp* | NM_028444.1 | 0.016 | 2.23 | 2.51 | protein kinase C, delta binding protein |
| *Sfxn4* | NM_053198 | 0.016 | 1.76 | 1.41 | sideroflexin 4 |
| *Rbp1* | NM_011254.2 | 0.016 | 2.24 | 1.85 | retinol binding protein 1 |
| *Plekhc1* | NM_146054.1 | 0.016 | 1.68 | 1.89 | pleckstrin homology domain containing, family C (with FERM domain) member 1 |
| *Ppp2r5d* | NM_009358.2 | 0.016 | 1.40 | 1.56 | protein phosphatase 2, regulatory subunit B (B56), delta isoform |
| *Dapp1* | NM_011932.1 | 0.016 | 1.26 | 1.27 | phosphotyrosine and 3-phosphoinositides 1 |
| *Entpd5* | NM_007647 | 0.016 | 1.58 | 1.48 | ectonucleoside triphosphate diphosphohydrolase 5 |
| *Nfatc1* | NM_198429.1 | 0.016 | 1.54 | 1.25 | nuclear factor of activated T-cells, cytoplasmic, calcineurin-dependent 1 |
| *Diras2* | NM_001024474.2 | 0.016 | 1.32 | 1.42 | DIRAS family, GTP-binding RAS-like 2 |
| *Ltap* | NM_033509 | 0.016 | 1.76 | 1.61 | vang-like 2 (van gogh, Drosophila) |
| *Klhl15* | NM_153165.2 | 0.016 | 1.07 | -1.26 | kelch-like 15 (Drosophila) |
| *Pcbd2* | NM_028281.1 | 0.016 | 1.85 | 1.76 | pterin 4 alpha carbinolamine dehydratase/dimerization cofactor of hepatocyte nuclear factor 1 alpha (TCF1) 2 |
| *Slc30a5* | NM_022885.1 | 0.016 | 1.43 | 1.53 | solute carrier family 30 (zinc transporter), member 5 |
| *Dpysl4* | NM_011993.2 | 0.016 | 4.42 | 3.28 | dihydropyrimidinase-like 4 |
| *Fem1b* | NM_010193.3 | 0.016 | 1.31 | 1.66 | feminization 1 homolog b (C. elegans) |
| *Igsf4a* | NM_018770 | 0.016 | 1.88 | 1.98 | immunoglobulin superfamily, member 4a |
| *Slco6c1* | XM_129976.2 | 0.016 | 1.19 | 1.23 | solute carrier organic anion transporter family, member 6c1 |
| *Acadsb* | NM_025826 | 0.016 | 1.37 | 1.44 | acyl-Coenzyme A dehydrogenase, short/branched chain |
| *S100a1* | NM_011309.2 | 0.016 | 3.17 | 3.00 | S100 calcium binding protein A1 |
| *Ssfa2* | NM_080558 | 0.016 | 1.56 | 2.33 | sperm specific antigen 2 |
| *Hspb1* | NM_013560 | 0.016 | 1.44 | 8.18 | heat shock protein 1 |
| *Slc29a3* | NM_023596.3 | 0.016 | 1.13 | -1.13 | solute carrier family 29 (nucleoside transporters), member 3 |
| *Swap70* | NM_009302.2 | 0.016 | 1.38 | 1.60 | SWA-70 protein |
| *Stx7* | NM_016797.2 | 0.017 | 1.30 | 1.29 | syntaxin 7 |
| *Sema3f* | NM_011349.2 | 0.017 | 1.73 | 1.84 | sema domain, immunoglobulin domain (Ig), short basic domain, secreted, (semaphorin) 3 F |
| *Idb3* | NM_008321.1 | 0.017 | 1.77 | 2.59 | inhibitor of DNA binding 3 |
| *Kif2a* | NM_008442.1 | 0.017 | 1.14 | 1.37 | kinesin family member 2A |
| *Osbpl3* | NM_027881.1 | 0.017 | 1.34 | 1.70 | oxysterol binding protein-like 3 |
| *Slc25a25* | NM_146118.2 | 0.017 | 1.23 | 1.66 | solute carrier family 25 (mitochondrial carrier, phosphate carrier), member 25 |
| *Pccb* | NM_025835.1 | 0.017 | 1.49 | 1.52 | propionyl Coenzyme A carboxylase, beta polypeptide |
| *Diras1* | NM_145217.2 | 0.017 | 1.95 | 1.53 | DIRAS family, GTP-binding RAS-like 1 |
| *Fgfrl1* | NM_054071.1 | 0.017 | 1.70 | 1.65 | fibroblast growth factor receptor-like 1 |
| *Gata2* | NM_008090.3 | 0.017 | 2.07 | 2.10 | GATA binding protein 2 |
| *Net1* | NM_019671 | 0.017 | 1.55 | 1.56 | neuroepithelial cell transforming gene 1 |
| *Prpf8* | NM_138659.1 | 0.017 | 1.00 | 1.36 | pre-mRNA processing factor 8 |
| *Rex3* | NM_009052.1 | 0.017 | 1.03 | 1.26 | reduced expression 3 |
| *Ttyh3* | NM_175274.2 | 0.017 | 2.41 | 2.07 | tweety homolog 3 (Drosophila) |
| *Rhou* | NM_133955.1 | 0.017 | 1.04 | 1.33 | ras homolog gene family, member U |
| *Schip1* | NM_013928.2 | 0.017 | 2.05 | 1.94 | schwannomin interacting protein 1 |
| *Scamp5* | NM_020270.2 | 0.017 | 1.43 | -1.01 | secretory carrier membrane protein 5 |
| *Zcchc9* | NM_145453.1 | 0.017 | 1.14 | 1.39 | zinc finger, CCHC domain containing 9 |
| *Slc35f1* | NM_178675.3 | 0.018 | 1.91 | 2.21 | solute carrier family 35, member F1 |
| *Rdh5* | NM_134006.3 | 0.018 | 1.76 | 1.73 | retinol dehydrogenase 5 |
| *Rbmx* | NM_011252 | 0.018 | 1.89 | 1.83 | RNA binding motif protein, X chromosome |
| *Tm4sf6* | NM_019656.2 | 0.018 | 1.71 | 1.59 | tetraspanin 6 |
| *Hist1h2bh* | NM_178197.1 | 0.018 | 1.98 | 2.02 | histone cluster 1, H2bh |
| *F11r* | NM_172647.1 | 0.018 | 1.78 | 1.83 | F11 receptor |
| *Nap1l4* | NM_008672 | 0.018 | 1.19 | 1.14 | nucleosome assembly protein 1-like 4 |
| *Rplp1* | NM_018853.3 | 0.018 | 1.32 | 1.46 | ribosomal protein, large, P1 |
| *Shh* | NM_009170 | 0.018 | 5.46 | 6.94 | sonic hedgehog |
| *Btbd6* | NM_201646.1 | 0.018 | 1.43 | 1.29 | BTB (POZ) domain containing 6 |
| *Foxd1* | NM_008242.1 | 0.018 | 1.29 | 1.66 | forkhead box D1 |
| *Fvt1* | NM_027534.1 | 0.018 | 1.10 | -1.06 | follicular lymphoma variant translocation 1 |
| *Gdap1l1* | NM_144891.1 | 0.018 | 1.64 | 1.51 | ganglioside-induced differentiation-associated protein 1-like 1 |
| *Rps26* | NM_013765.1 | 0.018 | 1.58 | 1.93 | ribosomal protein S26 |
| *Mtrf1l* | NM_175374.2 | 0.018 | 1.22 | 1.63 | mitochondrial translational release factor 1-like |
| *Catnbip1* | NM_023465.2 | 0.018 | 1.15 | 1.21 | catenin beta interacting protein 1 |
| *Clu* | NM_013492.1 | 0.018 | 2.38 | 2.09 | clusterin |
| *Slc39a12* | NM_001012305.1 | 0.018 | 4.33 | 3.15 | solute carrier family 39 (zinc transporter), member 12 |
| *Icam1* | NM_010493.2 | 0.018 | 1.59 | 1.55 | intercellular adhesion molecule |
| *Nes* | NM_016701.3 | 0.018 | 2.23 | 3.05 | nestin |
| *Sulf1* | NM_172294 | 0.018 | 2.76 | 2.67 | sulfatase 1 |
| *Smtn* | NM_013870.1 | 0.018 | 1.52 | 1.63 | smoothelin |
| *Srr* | NM_013761.2 | 0.018 | 1.38 | -1.12 | serine racemase |
| *Hs2st1* | NM_011828.2 | 0.018 | 1.68 | 1.44 | heparan sulfate 2-O-sulfotransferase 1 |
| *Per2* | NM_011066.1 | 0.019 | 1.52 | 1.55 | period homolog 2 (Drosophila) |
| *Cse1l* | NM_023565.1 | 0.019 | 1.17 | 1.36 | chromosome segregation 1-like (S. cerevisiae) |
| *Rbms1* | NM_020296.1 | 0.019 | 1.44 | 1.21 | RNA binding motif, single stranded interacting protein 1 |
| *Acot11* | NM_025590.3 | 0.019 | 4.03 | 3.07 | acyl-CoA thioesterase 11 |
| *Myadm* | NM_016969.1 | 0.019 | 1.68 | 1.53 | myeloid-associated differentiation marker |
| *Rbms2* | NM_019711.2 | 0.019 | 1.51 | 1.53 | RNA binding motif, single stranded interacting protein 2 |
| *Eef2* | NM_007907.1 | 0.019 | 1.48 | 1.43 | eukaryotic translation elongation factor 2 |
| *Nap1l1* | NM_015781.2 | 0.019 | 1.50 | 1.88 | nucleosome assembly protein 1-like 1 |
| *Sox7* | NM_011446.1 | 0.019 | 2.14 | 3.04 | SRY-box containing gene 7 |
| *Calm3* | NM_007590.2 | 0.019 | 1.49 | 1.37 | calmodulin 3 |
| *Snn* | NM_009223.2 | 0.019 | 1.01 | -1.33 | stannin |
| *Fgfr3* | NM_008010.2 | 0.019 | 1.69 | 1.76 | fibroblast growth factor 3 |
| *Ddah2* | NM_016765 | 0.019 | 1.24 | 1.18 | dimethylarginine dimethylaminohydrolase 2 |
| *Cyr61* | NM_010516.1 | 0.019 | 1.50 | 3.22 | cysteine rich protein 61 |
| *Arhgap20* | NM_175535.3 | 0.020 | 1.81 | 2.16 | Rho GTPase activating protein 20 |
| *Hc* | NM_010406.1 | 0.020 | 1.28 | 1.22 | hemolytic complement |
| *Socs3* | NM_007707.2 | 0.020 | 1.07 | 1.71 | suppressor of cytokine signaling 3 |
| *Tm4sf10* | NM_175771.2 | 0.020 | 2.22 | 2.06 | transmembrane protein 47 |
| *Dag1* | NM_010017.1 | 0.020 | 1.60 | 1.51 | dystroglycan 1 |
| *Lpxn* | NM_134152.1 | 0.020 | 1.12 | 1.11 | leupaxin |
| *Cdc2l5* | NM_001081058.1 | 0.020 | 1.08 | 1.22 | cell division cycle 2-like 5 (cholinesterase-related cell division controller) |
| *Phldb1* | NM_153537.3 | 0.020 | 1.21 | 1.43 | pleckstrin homology-like domain, family B, member 1 |
| *Apobec1* | NM_031159.2 | 0.020 | 2.46 | 1.91 | apolipoprotein B editing complex 1 |
| *Muted* | NM_139063.1 | 0.020 | 1.57 | 1.26 | muted |
| *Tbx3* | NM_011535.2 | 0.020 | 3.00 | 3.06 | T-box 3 |
| *Hmgn3* | NM_175074.1 | 0.020 | 1.83 | 1.83 | high mobility group nucleosomal binding domain 3 |
| *Arrdc3* | NM_178917.2 | 0.020 | 2.82 | 1.67 | arrestin domain containing 3 |
| *Camk2g* | NM_178597.2 | 0.020 | 1.25 | 1.38 | calcium/calmodulin-dependent protein kinase II gamma |
| *Efna4* | NM_007910.1 | 0.020 | 1.09 | -1.16 | ephrin A4 |
| *Noc4* | NM_010926.1 | 0.020 | 1.46 | 1.48 | COX4 neighbor |
| *Samd4* | NM_001037221.1 | 0.020 | 1.44 | 1.48 | sterile alpha motif domain containing 4 |
| *Slc16a6* | NM_134038 | 0.020 | 1.62 | 1.34 | solute carrier family 16 (monocarboxylic acid transporters), member 6 |
| *Myh10* | NM_175260 | 0.020 | 2.87 | 3.90 | myosin, heavy polypeptide 10, non-muscle |
| *Slc20a2* | NM_011394.1 | 0.020 | 1.23 | -1.17 | solute carrier family 20, member 2 |
| *Kcnk1* | NM_008430.1 | 0.020 | 2.32 | 1.90 | potassium channel, subfamily K, member 1 |
| *Snag1* | NM_130796.1 | 0.020 | 1.72 | 2.23 | sorting nexin associated golgi protein 1 |
| *Anapc2* | NM_175300.2 | 0.021 | 1.38 | 1.26 | anaphase promoting complex subunit 2 |
| *Kif18a* | NM_139303.1 | 0.021 | 1.34 | 1.32 | kinesin family member 18A |
| *Plekha2* | NM_031257.2 | 0.021 | 1.46 | 1.51 | pleckstrin homology domain-containing, family A (phosphoinositide binding specific) member 2 |
| *Abcc5* | NM_176839.1 | 0.021 | 1.73 | 1.42 | ATP-binding cassette, sub-family C (CFTR/MRP), member 5 |
| *Ankrd22* | NM_024204.4 | 0.021 | 1.10 | 1.04 | ankyrin repeat domain 22 |
| *Aqp11* | NM_175105.2 | 0.021 | 1.44 | 1.43 | aquaporin 11 |
| *Jarid1d* | NM_011419.1 | 0.021 | 1.80 | 1.84 | jumonji, AT rich interactive domain 1 |
| *Lgals8* | NM_018886.2 | 0.021 | 1.44 | 1.27 | lectin, galactose binding, soluble 8 |
| *Rps2* | NM_008503.2 | 0.021 | 1.43 | 1.59 | ribosomal protein S2 |
| *Sh3yl1* | NM_013709.2 | 0.021 | 1.59 | 1.50 | Sh3 domain YSC-like 1 |
| *Rab5c* | NM_024456 | 0.021 | 1.16 | 1.24 | RAB5C, member RAS oncogene family |
| *Rab6b* | NM_173781.3 | 0.021 | 1.25 | 1.12 | RAB6B, member RAS oncogene family |
| *Emd* | NM_007927.1 | 0.021 | 1.39 | 1.37 | emerin |
| *Fgfr2* | NM_201601.1 | 0.021 | 1.79 | 1.82 | fibroblast growth factor 2 |
| *Msx1* | NM_010835.1 | 0.021 | 2.28 | 2.71 | homeo box, msh-like 1 |
| *Peci* | NM_011868.1 | 0.021 | 1.55 | 1.65 | peroxisomal delta3, delta2-enoyl-Coenzyme A isomerase |
| *Tsga2* | NM_025290.2 | 0.021 | 1.86 | 1.58 | testis specific gene A2 |
| *Anxa6* | NM_013472.2 | 0.022 | 1.75 | 1.92 | annexin A6 |
| *Gadd45b* | NM_008655.1 | 0.022 | 1.86 | 2.76 | growth arrest and DNA-damage-inducible 45 beta |
| *Ppp1r9a* | NM_181595.2 | 0.022 | 1.24 | 1.06 | protein phosphatase 1, regulatory (inhibitor) subunit 9A |
| *Gpx1* | NM_008160.1 | 0.022 | 1.31 | 1.45 | glutathione peroxidase 1 |
| *AF322649* | NM_153134.2 | 0.022 | 1.52 | 1.49 | immunity-related GTPase family, Q |
| *Mto1* | NM_026658.1 | 0.022 | 1.02 | 1.25 | mitochondrial translation optimization 1 homolog (S. cerevisiae) |
| *Fbxo3* | NM_212433.1 | 0.022 | 1.42 | 1.39 | F-box protein 3 |
| *Tspan17* | NM_028841.1 | 0.022 | 1.37 | 1.12 | tetraspanin 17 |
| *Pdlim2* | NM_145978.1 | 0.022 | 1.54 | 1.54 | PDZ and LIM domain 2 |
| *Nusap1* | NM_133851 | 0.023 | 1.69 | 1.55 | nucleolar and spindle associated protein 1 |
| *Ppm1d* | NM_016910.2 | 0.023 | 1.19 | 1.02 | protein phosphatase 1D magnesium-dependent, delta isoform |
| *Nrn1* | NM_153529.1 | 0.023 | 1.43 | 1.66 | neuritin 1 |
| *P4ha1* | NM_011030.1 | 0.023 | 1.18 | 1.91 | procollagen-proline, 2-oxoglutarate 4-dioxygenase (proline 4-hydroxylase), alpha 1 polypeptide |
| *Rfxap* | NM_133231 | 0.023 | 1.64 | 1.93 | regulatory factor X-associated protein |
| *Sox3* | NM_009237.1 | 0.023 | 1.11 | 1.17 | SRY-box containing gene 3 |
| *Nrarp* | NM_025980.1 | 0.023 | 1.83 | 1.84 | Notch-regulated ankyrin repeat protein |
| *Abhd7* | NM_001001804.1 | 0.023 | 1.71 | 1.49 | abhydrolase domain containing 7 |
| *Ltk* | NM_203345.1 | 0.023 | 1.37 | 1.25 | leukocyte tyrosine kinase |
| *Slc2a10* | NM_130451.1 | 0.023 | 1.58 | 1.41 | solute carrier family 2 (facilitated glucose transporter), member 10 |
| *Kctd15* | NM_146188.1 | 0.023 | 1.18 | 1.31 | potassium channel tetramerisation domain containing 15 |
| *Heca* | NM_001033432.1 | 0.023 | 1.15 | -1.14 | headcase homolog (Drosophila) |
| *Slc35b1* | XM_128634.4 | 0.023 | 1.25 | 1.05 | solute carrier family 35, member B1 |
| *Hebp2* | NM_019487.2 | 0.023 | 1.79 | 1.52 | heme binding protein 2 |
| *Baalc* | NM_080640 | 0.023 | 1.94 | 1.63 | brain and acute leukemia, cytoplasmic |
| *Dscr1l2* | NM_022980.3 | 0.023 | 1.32 | 1.16 | Down syndrome critical region gene 1-like 2 |
| *Cdsn* | NM_001008424.2 | 0.023 | 1.72 | 1.61 | corneodesmosin |
| *Nrcam* | NM_176930.2 | 0.023 | 1.20 | 1.21 | neuron-glia-CAM-related cell adhesion molecule |
| *Gng5* | NM_010318.2 | 0.023 | 1.27 | 1.44 | guanine nucleotide binding protein (G protein), gamma 5 subunit |
| *Myl6* | NM_010860.2 | 0.023 | 1.87 | 1.86 | myosin, light polypeptide 6, alkali, smooth muscle and non-muscle |
| *Nvl* | NM_026171.1 | 0.023 | 1.05 | 1.32 | nuclear VCP-like |
| *Pop4* | NM_025390 | 0.023 | 1.78 | 1.81 | processing of precursor 4, ribonuclease P/MRP family, (S. cerevisiae) |
| *Mmp11* | NM_008606.1 | 0.024 | 1.71 | 1.52 | matrix metallopeptidase 11 |
| *Lonp2* | NM_025827.2 | 0.024 | 1.33 | 1.12 | lon peptidase 2, peroxisomal |
| *Biklk* | NM_007546.1 | 0.024 | 1.68 | 1.30 | Bcl2-interacting killer |
| *Myl4* | NM_010858.3 | 0.024 | 1.70 | 1.44 | myosin, light polypeptide 4 |
| *Rage* | NM_011973 | 0.024 | 1.50 | 1.28 | serine/threonine kinase 30 |
| *Hn1l* | NM_198937.2 | 0.024 | 1.49 | 1.75 | hematological and neurological expressed 1-like |
| *Epn2* | NM_010148 | 0.024 | 1.01 | 1.35 | epsin 2 |
| *Asb7* | NM_178236.2 | 0.024 | 1.15 | 1.45 | ankyrin repeat and SOCS box-containing protein 7 |
| *Fto* | NM_011936.1 | 0.024 | 1.08 | -1.12 | fat mass and obesity associated |
| *Gfap* | NM_010277 | 0.024 | 2.02 | 1.85 | glial fibrillary acidic protein |
| *Mns1* | NM_008613.1 | 0.024 | 1.46 | 1.47 | meiosis-specific nuclear structural protein 1 |
| *Psmd12* | NM_025894.1 | 0.024 | 1.01 | 1.16 | proteasome (prosome, macropain) 26S subunit, non-ATPase, 12 |
| *Sparc* | NM_009242.1 | 0.024 | 1.50 | 1.59 | secreted acidic cysteine rich glycoprotein |
| *Ssrp1* | NM_182990.2 | 0.024 | 1.11 | 1.25 | structure specific recognition protein 1 |
| *Efnb1* | NM_010110.2 | 0.024 | 1.75 | 1.68 | ephrin B1 |
| *Scara3* | NM_172604.1 | 0.024 | 2.07 | 1.47 | scavenger receptor class A, member 3 |
| *Prc1* | NM_145150.1 | 0.024 | 1.48 | 1.44 | protein regulator of cytokinesis 1 |
| *Trip10* | NM_134125.1 | 0.024 | 1.10 | 1.32 | thyroid hormone receptor interactor 10 |
| *Sept9* | NM_017380.1 | 0.024 | 1.84 | 2.07 | septin 9 |
| *Cryz* | NM_009968.1 | 0.024 | 1.45 | 1.08 | crystallin, zeta |
| *Kif22* | NM_145588.1 | 0.024 | 1.63 | 1.25 | kinesin family member 22 |
| *Aard* | NM_175503.2 | 0.024 | 3.06 | 2.66 | alanine and arginine rich domain containing protein |
| *Ift140* | NM_134126.2 | 0.024 | 1.22 | -1.05 | intraflagellar transport 140 homolog (Chlamydomonas) |
| *Nras* | NM_010937.2 | 0.024 | 1.00 | -1.19 | neuroblastoma ras oncogene |
| *Papss1* | NM_011863.1 | 0.024 | 1.48 | 1.39 | 3'-phosphoadenosine 5'-phosphosulfate synthase 1 |
| *Hoxa5* | NM_010453.2 | 0.025 | 1.87 | 1.96 | homeo box A5 |
| *Tspan7* | NM_019634.2 | 0.025 | 2.82 | 2.20 | tetraspanin 7 |
| *Ednrb* | NM_007904.2 | 0.025 | 5.32 | 3.42 | endothelin receptor type B |
| *Adnp* | NM_009628.2 | 0.025 | 1.22 | 1.21 | activity-dependent neuroprotective proteiN |
| *Ltbp4* | NM_175641.1 | 0.025 | 2.07 | 2.14 | latent transforming growth factor beta binding protein 4 |
| *Btbd14a* | NM_001037098.1 | 0.025 | 2.73 | 2.46 | BTB (POZ) domain containing 14A |
| *Cryl1* | NM_030004.2 | 0.025 | 2.30 | 1.88 | crystallin, lambda 1 |
| *Atoh8* | NM_153778.2 | 0.025 | 1.06 | 1.43 | atonal homolog 8 (Drosophila) |
| *Plk1* | NM_011121.2 | 0.025 | 1.56 | 1.39 | pleckstrin homology domain containing, family G (with RhoGef domain) member 2 |
| *Rap1gds1* | NM_145544 | 0.025 | 1.25 | 1.32 | RAP1, GTP-GDP dissociation stimulator 1 |
| *Ap3m2* | NM_029505.1 | 0.025 | 1.35 | 1.43 | adaptor-related protein complex 3, mu 2 subunit |
| *Arhgap22* | NM_153800.2 | 0.025 | 2.06 | 1.96 | Rho GTPase activating protein 22 |
| *Mif4gd* | NM_027162.3 | 0.025 | 1.28 | 1.14 | MIF4G domain containing |
| *Sbk* | NM_145587.1 | 0.025 | 1.44 | 1.07 | SH3-binding kinase 1 |
| *Hist1h1c* | NM_015786.1 | 0.025 | 2.24 | 1.85 | histone cluster 1, H1c |
| *Zfp96* | NM_016684.1 | 0.025 | 1.24 | 1.09 | zinc finger protein 96 |
| *Sparc* | NM_009242.1 | 0.025 | 1.51 | 1.55 | secreted acidic cysteine rich glycoprotein |
| *Cish* | NM_009895.2 | 0.025 | 1.41 | 1.23 | cytokine inducible SH2-containing protein |
| *Eif4g2* | NM_013507.2 | 0.025 | 1.19 | 1.32 | eukaryotic translation initiation factor 4, gamma 2 |
| *Gyg1* | NM_013755.1 | 0.025 | 1.47 | 1.66 | glycogenin |
| *Vpreb2* | NM_016983.1 | 0.025 | 1.11 | 1.24 | pre-B lymphocyte gene 2 |
| *Mettl9* | NM_021554.2 | 0.026 | 1.34 | 1.30 | methyltransferase like 9 |
| *Cetn4* | NM_145825.1 | 0.026 | 1.72 | 1.60 | centrin 4 |
| *Cyb561* | NM_007805.2 | 0.026 | 1.27 | 1.29 | cytochrome b-561 |
| *Gpc3* | NM_016697.2 | 0.026 | 1.49 | 1.50 | glypican 3 |
| *Gemin7* | NM_027189.1 | 0.026 | 1.23 | 1.36 | gem (nuclear organelle) associated protein 7 |
| *Igsf9b* | NM_001033323.1 | 0.026 | 1.02 | 1.18 | immunoglobulin superfamily, member 9B |
| *Lix1* | NM_025681 | 0.026 | 1.50 | 1.29 | limb expression 1 homolog |
| *Nupr1* | NM_019738.1 | 0.026 | 1.16 | 1.91 | nuclear protein 1 |
| *Stx6* | NM_021433.2 | 0.026 | 1.18 | 1.20 | syntaxin 6 |
| *Axin2* | NM_015732.3 | 0.026 | 1.40 | 1.92 | axin2 |
| *Mkrn3* | NM_011746.1 | 0.026 | 1.81 | 1.77 | makorin, ring finger protein, 3 |
| *Hnrpc* | NM_016884 | 0.026 | 1.32 | 1.38 | heterogeneous nuclear ribonucleoprotein C |
| *Kctd5* | NM_027008.1 | 0.026 | 1.43 | 1.67 | potassium channel tetramerisation domain containing 5 |
| *Nlrx1* | NM_178420.2 | 0.026 | 1.65 | 1.26 | NLR family member X1 |
| *Abcd3* | NM_008991.2 | 0.026 | 1.46 | 1.38 | ATP-binding cassette, sub-family D (ALD), member 3 |
| *Lrrc5* | NM_178701.2 | 0.026 | 1.05 | -1.16 | leucine rich repeat containing 8D |
| *Nfix* | NM_010906.1 | 0.026 | 1.72 | 1.39 | nuclear factor I/X |
| *Pcbp3* | NM_021568.1 | 0.026 | 1.72 | 1.73 | poly(rC) binding protein 3 |
| *Csnk1d* | NM_027874.1 | 0.026 | 1.43 | 1.24 | casein kinase 1, delta |
| *Obfc2b* | NM_027257.1 | 0.026 | 1.74 | 1.80 | oligonucleotide/oligosaccharide-binding fold containing 2B |
| *Zfp639* | NM_144519.2 | 0.027 | 1.31 | 1.58 | zinc finger protein 639 |
| *Ivd* | NM_019826 | 0.027 | 1.59 | 1.54 | isovaleryl coenzyme A dehydrogenase |
| *Fnbp4* | NM_018828.1 | 0.027 | 1.06 | 1.10 | formin binding protein 4 |
| *Fkbp7* | NM_010222.1 | 0.027 | 1.40 | 1.44 | FK506 binding protein 7 |
| *Hrsp12* | NM_008287.2 | 0.027 | 1.58 | 1.35 | heat-responsive protein 12 |
| *Lrfn3* | NM_175478.2 | 0.027 | 1.45 | 1.06 | leucine rich repeat and fibronectin type III domain containing 3 |
| *Mical1* | NM_138315.1 | 0.027 | 2.09 | 1.89 | microtubule associated monoxygenase, calponin and LIM domain containing 1 |
| *Tpd52* | NM_009412.2 | 0.027 | 1.48 | 1.25 | tumor protein D52 |
| *Itga11* | NM_176922.4 | 0.027 | 1.62 | 1.87 | integrin, alpha 11 |
| *Colm* | NM_177350.2 | 0.027 | 2.12 | 2.32 | gliomedin |
| *Leng9* | NM_175529.2 | 0.027 | 1.13 | -1.20 | leukocyte receptor cluster (LRC) member 9 |
| *Hdgf* | NM_008231.2 | 0.027 | 1.23 | 1.16 | hepatoma-derived growth factor |
| *Traf4* | NM_009423.2 | 0.027 | 1.11 | 1.47 | Tnf receptor associated factor 4 |
| *Rpl13a* | NM_009438 | 0.028 | 1.71 | 2.24 | ribosomal protein L13A |
| *Plekhg2* | NM_138752.1 | 0.028 | 1.25 | 1.35 | pleckstrin homology domain containing, family G (with RhoGef domain) member 2 |
| *Galnt11* | NM_144908.1 | 0.028 | 1.36 | -1.15 | UDP-N-acetyl-alpha-D-galactosamine:polypeptide N-acetylgalactosaminyltransferase 11 |
| *Arl6ip1* | NM_019419.1 | 0.028 | 1.55 | 1.46 | ADP-ribosylation factor-like 6 interacting protein 1 |
| *Luc7l* | NM_028190.1 | 0.028 | 1.08 | 1.70 | Luc7 homolog (S. cerevisiae)-like |
| *Ntn4* | NM_021320.2 | 0.028 | 2.34 | 2.01 | netrin 4 |
| *Slc16a4* | NM_146136.1 | 0.028 | 1.89 | 1.82 | solute carrier family 16 (monocarboxylic acid transporters), member 4 |
| *Mnd1* | NM_029797.1 | 0.028 | 1.80 | 1.69 | meiotic nuclear divisions 1 homolog (S. cerevisiae) |
| *Llgl1* | NM_008502.1 | 0.028 | 1.03 | -1.12 | lethal giant larvae homolog 1 (Drosophila) |
| *Pex6* | NM_145488.1 | 0.028 | 1.42 | 1.33 | peroxisomal biogenesis factor 6 |
| *Twist1* | NM_011658.1 | 0.028 | 1.51 | 1.97 | twist gene homolog 1 |
| *Heph* | NM_010417.1 | 0.029 | 1.50 | 1.35 | hephaestin |
| *Plekhe1* | NM_133821.1 | 0.029 | 1.21 | 1.35 | pleckstrin homology domain containing, family E (with leucine rich repeats) member 1 |
| *Dusp2* | NM_010090.2 | 0.029 | 1.10 | 1.40 | dual specificity phosphatase 2 |
| *Bckdk* | NM_009739.2 | 0.029 | 1.40 | 1.19 | branched chain ketoacid dehydrogenase kinase |
| *Cd200* | NM_010818 | 0.029 | 1.89 | 1.44 | CD200 antigen |
| *Dnajc7* | NM_019795.3 | 0.029 | 1.56 | 1.34 | DnaJ (Hsp40) homolog, subfamily C, member 7 |
| *Guk1* | NM_008193.2 | 0.030 | 1.67 | 1.46 | guanylate kinase 1 |
| *Slc2a1* | NM_011400.1 | 0.030 | 1.85 | 1.98 | solute carrier family 2 (facilitated glucose transporter), member 1 |
| *Sfxn5* | NM_178639.2 | 0.030 | 2.69 | 2.25 | sideroflexin 5 |
| *Sri* | NM_001080974.1 | 0.030 | 1.38 | 1.27 | sorcin |
| *Eef1b2* | NM_018796.2 | 0.030 | 1.47 | 1.81 | eukaryotic translation elongation factor 1 beta 2 |
| *Ptpla* | NM_013935.1 | 0.030 | 1.22 | 1.46 | protein tyrosine phosphatase-like (proline instead of catalytic arginine), member a |
| *Zfp98* | NM_016793.1 | 0.030 | 1.82 | 1.52 | zinc finger protein 99 |
| *Lmod1* | NM_053106.1 | 0.030 | 1.95 | 1.98 | leiomodin 1 (smooth muscle) |
| *Aprt* | NM_009698.1 | 0.030 | 2.49 | 2.56 | adenine phosphoribosyl transferase |
| *Efcab2* | NM_026626.2 | 0.030 | 1.17 | 1.18 | EF-hand calcium binding domain 2 |
| *Adipor1* | NM_028320.2 | 0.030 | 1.19 | 1.20 | adiponectin receptor 1 |
| *Clps* | NM_025469.1 | 0.030 | 1.07 | 1.03 | colipase, pancreatic |
| *Epb4.1l2* | NM_013511 | 0.030 | 2.41 | 2.47 | erythrocyte protein band 4.1-like 2 |
| *Wdr47* | NM_181400.2 | 0.030 | 1.23 | 1.39 | WD repeat domain 47 |
| *Atp1a1* | NM_144900.1 | 0.030 | 1.25 | 1.37 | ATPase, Na+/K+ transporting, alpha 1 polypeptide |
| *Cav1* | NM_007616.2 | 0.030 | 2.88 | 2.88 | caveolin, caveolae protein 1 |
| *Exosc8* | NM_027148.2 | 0.030 | 1.18 | 1.27 | exosome component 8 |
| *Nr2c1* | NM_011629.2 | 0.030 | 1.11 | 1.19 | nuclear receptor subfamily 2, group C, member 1 |
| *Olfm2* | NM_173777.2 | 0.030 | 1.43 | 1.31 | olfactomedin 2 |
| *Smr1* | NM_011422.1 | 0.030 | 1.04 | 1.17 | submaxillary gland androgen regulated protein 1 |
| *Snx3* | NM_017472.2 | 0.030 | 1.24 | 1.48 | sorting nexin 3 |
| *Ap2s1* | NM_198613.1 | 0.030 | 1.46 | 1.49 | adaptor-related protein complex 2, sigma 1 subunit |
| *Ube2e3* | NM_009454.2 | 0.030 | 1.17 | 1.13 | ubiquitin-conjugating enzyme E2E 3, UBC4/5 homolog (yeast) |
| *Lrp2* | NM_001081088.1 | 0.030 | 1.03 | 1.23 | low density lipoprotein receptor-related protein 2 |
| *Heg1* | NM_175256.4 | 0.031 | 1.17 | 1.28 | HEG homolog 1 (zebrafish) |
| *Fah* | NM_010176.1 | 0.031 | 2.06 | 1.98 | fumarylacetoacetate hydrolase |
| *Zfp60* | NM_029531.2 | 0.031 | 1.33 | 1.64 | zinc finger protein 60 |
| *Tbc1d2b* | NM_194334.2 | 0.031 | 1.80 | 2.20 | TBC1 domain family, member 2B |
| *Pmp22* | NM_008885.1 | 0.031 | 1.51 | -1.02 | peripheral myelin protein |
| *Mgll* | NM_011844.3 | 0.031 | 2.98 | 2.24 | monoglyceride lipase |
| *Slc25a38* | NM_144793.1 | 0.031 | 1.27 | 1.64 | solute carrier family 25, member 38 |
| *Vcam1* | NM_011693.2 | 0.031 | 1.49 | 1.87 | vascular cell adhesion molecule 1 |
| *Cpne8* | NM_001033851.1 | 0.031 | 1.79 | 1.63 | copine VIII |
| *Slc1a4* | NM_018861.2 | 0.031 | 1.44 | 1.71 | solute carrier family 1 (glutamate/neutral amino acid transporter), member 4 |
| *Msi2h* | NM_054043 | 0.031 | 1.82 | 1.76 | Musashi homolog 2 |
| *Il7* | NM_008371.2 | 0.031 | 1.57 | 1.56 | interleukin 7 |
| *Vrk3* | NM_133945.1 | 0.031 | 1.38 | 1.17 | vaccinia related kinase 3 |
| *Slc9a3r2* | NM_023055.1 | 0.031 | 1.77 | 1.49 | solute carrier family 9 (sodium/hydrogen exchanger), isoform 3 regulator 2 |
| *Arfip2* | NM_029802.2 | 0.031 | 1.19 | 1.10 | ADP-ribosylation factor interacting protein 2 |
| *Ube2n* | NM_080560.2 | 0.031 | 1.42 | 1.54 | ubiquitin-conjugating enzyme E2N |
| *Sirt5* | NM_178848.2 | 0.032 | 1.41 | 1.29 | sirtuin 5 (silent mating type information regulation 2 homolog) 5 |
| *Thbs3* | NM_013691.1 | 0.032 | 1.55 | 1.54 | thrombospondin 3 |
| *Tnfaip2* | NM_009396.1 | 0.032 | 1.50 | 1.28 | tumor necrosis factor, alpha-induced protein 2 |
| *Ntrk2* | NM_001025074.1 | 0.032 | 4.45 | 3.77 | neurotrophic tyrosine kinase, receptor, type 2 |
| *Pfkl* | NM_008826.2 | 0.032 | 1.68 | 1.67 | phosphofructokinase, liver, B-type |
| *Zfp207* | NM_011751.1 | 0.032 | 1.19 | 1.35 | zinc finger protein 207 |
| *Mxi1* | NM_010847.1 | 0.032 | 1.19 | 1.25 | Max interacting protein 1 |
| *Il18* | NM_008360.1 | 0.032 | 2.03 | 2.08 | interleukin 18 |
| *Palm* | NM_023128.2 | 0.032 | 1.44 | 1.28 | paralemmin |
| *Nphp1* | NM_016902.1 | 0.032 | 1.52 | 1.40 | nephronophthisis 1 (juvenile) homolog (human) |
| *Slc12a8* | NM_134251 | 0.032 | 1.45 | 1.32 | solute carrier family 12 (potassium/chloride transporters), member 8 |
| *Inpp5f* | NM_178641.3 | 0.032 | 1.28 | 1.21 | inositol polyphosphate-5-phosphatase F |
| *Aatk* | NM_007377.1 | 0.032 | 1.86 | 1.68 | apoptosis-associated tyrosine kinase |
| *Ang1* | NM_007447.2 | 0.032 | 3.26 | 3.20 | angiogenin, ribonuclease, RNase A family, 5 |
| *Parva* | NM_020606.4 | 0.032 | 1.58 | 1.19 | parvin, alpha |
| *G6pc* | NM_008061.2 | 0.032 | 1.01 | 1.08 | glucose-6-phosphatase, catalytic |
| *Kcnj6* | NM_001025590.1 | 0.032 | 1.01 | 1.10 | potassium inwardly-rectifying channel, subfamily J, member 6 |
| *Ulk2* | NM_013881.3 | 0.032 | 1.45 | 1.57 | Unc-51 like kinase 2 (C. elegans) |
| *Arhgdig* | NM_008113.3 | 0.032 | 1.34 | 1.59 | Rho GDP dissociation inhibitor (GDI) gamma |
| *Zfp574* | NM_175477.2 | 0.032 | 1.26 | 1.55 | zinc finger protein 574 |
| *Supt4h* | NM_009296 | 0.033 | 1.49 | 1.44 | suppressor of Ty 4 homolog 1 |
| *Acvr1* | NM_007394 | 0.033 | 1.52 | 1.68 | activin A receptor, type 1 |
| *Bag3* | NM_013863.3 | 0.033 | 1.76 | 2.57 | Bcl2-associated athanogene 3 |
| *Hist1h2bc* | NM_023422.1 | 0.033 | 1.64 | 1.96 | histone 1, H2bc |
| *Zfp817* | NM_001007575.1 | 0.033 | 1.12 | 1.34 | zinc finger protein 817 |
| *Sin3a* | NM_011378 | 0.033 | 1.25 | -1.16 | transcriptional regulator, SIN3A (yeast) |
| *Rnf125* | NM_026301.1 | 0.033 | 1.63 | 1.39 | ring finger protein 125 |
| *Rgs10* | NM_026418.1 | 0.033 | 1.41 | 1.34 | regulator of G-protein signalling 10 |
| *Ppp6c* | NM_024209 | 0.033 | 1.65 | 1.54 | protein phosphatase 6, catalytic subunit |
| *Depdc1b* | NM_178683 | 0.033 | 1.36 | 1.58 | DEP domain containing 1B |
| *Sox8* | NM_011447.1 | 0.033 | 1.37 | 1.69 | SRY-box containing gene 8 |
| *Isca1* | NM_026921.2 | 0.033 | 1.57 | 1.56 | iron-sulfur cluster assembly 1 homolog (S. cerevisiae) |
| *Rgs7* | NM_011880.1 | 0.034 | 2.57 | 2.05 | regulator of G protein signaling 7 |
| *Cirbp* | NM_007705.1 | 0.034 | 1.81 | 1.03 | cold inducible RNA binding protein |
| *Slc27a3* | XM_130954.3 | 0.034 | 1.23 | 1.34 | solute carrier family 27 |
| *Wipi1* | NM_145940.2 | 0.034 | 1.28 | -1.07 | WD repeat domain, phosphoinositide interacting 1 |
| *Hist1h3d* | NM_178204 | 0.034 | 1.36 | 1.81 | histone cluster 1, H3d |
| *Rps3* | NM_012052.1 | 0.034 | 1.84 | 2.31 | ribosomal protein S3 |
| *Sox7* | NM_011446.1 | 0.034 | 1.61 | 2.13 | SRY-box containing gene 7 |
| *Rbpms* | NM_001042675.1 | 0.034 | 1.95 | 2.15 | RNA binding protein gene with multiple splicing |
| *Cdc23* | NM_178347 | 0.034 | 1.81 | 1.60 | cell division cycle 23 |
| *Psmd13* | NM_011875.2 | 0.034 | 1.32 | 1.22 | proteasome (prosome, macropain) 26S subunit, non-ATPase, 13 |
| *Vars2l* | NM_175137 | 0.034 | 1.01 | -1.09 | valyl-tRNA synthetase 2, mitochondrial (putative) |
| *Tbc1d22a* | NM_145476.2 | 0.034 | 1.02 | -1.29 | TBC1 domain family, member 22a |
| *Mfn1* | NM_024200.2 | 0.034 | 1.15 | 1.01 | mitofusin 1 |
| *Apoa2* | NM_013474.1 | 0.034 | 1.14 | 1.22 | apolipoprotein A-II |
| *Fst* | NM_008046.1 | 0.034 | 1.61 | 1.82 | follistatin |
| *Mocs2* | NM_013826.1 | 0.034 | 1.59 | 1.61 | molybdenum cofactor synthesis 2 |
| *Dnaja4* | NM_021422.2 | 0.034 | 1.04 | 1.58 | DnaJ (Hsp40) homolog, subfamily A, member 4 |
| *Rhobtb3* | NM_028493.1 | 0.034 | 1.68 | 1.71 | Rho-related BTB domain containing 3 |
| *Brd9* | NM_001024508.2 | 0.034 | 1.40 | 1.18 | bromodomain containing 9 |
| *Nedd9* | NM_017464.2 | 0.035 | 1.45 | 1.81 | neural precursor cell expressed, developmentally down-regulated gene 9 |
| *Nqo2* | NM_020282.2 | 0.035 | 1.87 | 1.85 | NAD(P)H dehydrogenase, quinone 2 |
| *Aadacl1* | NM_178772.2 | 0.035 | 1.39 | 1.39 | arylacetamide deacetylase-like 1 |
| *Pex19* | NM_023041.2 | 0.035 | 1.31 | 1.32 | peroxisome biogenesis factor 19 |
| *Ndufc1* | NM_025523.1 | 0.035 | 1.39 | 1.57 | NADH dehydrogenase (ubiquinone) 1, subcomplex unknown, 1 |
| *Nfe2l2* | NM_010902.2 | 0.035 | 1.15 | -1.16 | nuclear factor, erythroid derived 2, like 2 |
| *Ubfd1* | NM_138589 | 0.035 | 1.33 | 1.55 | ubiquitin family domain containing 1 |
| *Gnaz* | NM_010311.2 | 0.035 | 1.75 | 1.40 | guanine nucleotide binding protein, alpha z subunit |
| *Lig1* | NM_010715.1 | 0.035 | 1.25 | 1.00 | ligase I, DNA, ATP-dependent |
| *Pde6d* | NM_008801.2 | 0.035 | 1.15 | 1.31 | phosphodiesterase 6D, cGMP-specific, rod, delta |
| *Aplp2* | NM_009691.1 | 0.035 | 1.27 | 1.06 | amyloid beta (A4) precursor-like protein 2 |
| *Sars2* | NM_023637.1 | 0.035 | 1.20 | 1.23 | seryl-aminoacyl-tRNA synthetase 2 |
| *Adprtl2* | NM_009632.2 | 0.035 | 1.38 | 1.27 | poly (ADP-ribose) polymerase family, member 2 |
| *Dcun1d3* | NM_173408.1 | 0.035 | 1.07 | 1.46 | DCN1, defective in cullin neddylation 1, domain containing 3 (S. cerevisiae) |
| *Pts* | NM_011220.2 | 0.035 | 1.58 | 1.50 | 6-pyruvoyl-tetrahydropterin synthase |
| *Mmd2* | NM_175217.3 | 0.035 | 2.51 | 1.84 | monocyte to macrophage differentiation-associated 2 |
| *Bckdha* | NM_007533.2 | 0.035 | 1.28 | 1.02 | branched chain ketoacid dehydrogenase E1, alpha polypeptide |
| *Gapvd1* | NM_025709.2 | 0.035 | 1.13 | 1.16 | GTPase activating protein and VPS9 domains 1 |
| *Uqcrb* | NM_026219.1 | 0.035 | 1.37 | 1.58 | ubiquinol-cytochrome c reductase binding protein |
| *Kif15* | NM_010620.1 | 0.035 | 1.84 | 1.80 | kinesin family member 15 |
| *Ndc80* | NM_023294.1 | 0.035 | 1.23 | 1.33 | NDC80 homolog, kinetochore complex component (S. cerevisiae) |
| *Tcf7l2* | NM_009333.2 | 0.035 | 1.35 | 1.49 | transcription factor 7-like 2, T-cell specific, HMG-box |
| *Hint3* | NM_025798.2 | 0.035 | 1.42 | 1.52 | histidine triad nucleotide binding protein 3 |
| *Artn* | NM_009711.2 | 0.035 | 1.74 | 1.90 | artemin |
| *Slc30a6* | NM_144798.2 | 0.035 | 1.16 | 1.18 | solute carrier family 30 (zinc transporter), member 6 |
| *Uaca* | NM_028283 | 0.035 | 1.69 | 1.38 | uveal autoantigen with coiled-coil domains and ankyrin repeats |
| *Raly* | NM_023130.1 | 0.035 | 1.04 | 1.26 | hnRNP-associated with lethal yellow |
| *Cops5* | NM_013715.1 | 0.035 | 1.33 | 1.42 | COP9 (constitutive photomorphogenic) homolog, subunit 5 (Arabidopsis thaliana) |
| *Lace1* | NM_145743.1 | 0.035 | 1.28 | 1.24 | lactation elevated 1 |
| *Cyp39a1* | NM_018887.2 | 0.035 | 1.39 | 1.09 | cytochrome P450, family 39, subfamily a, polypeptide 1 |
| *Gbas* | NM_008095.1 | 0.036 | 1.01 | -1.30 | glioblastoma amplified sequence |
| *Ogn* | NM_008760.2 | 0.036 | 1.96 | 1.56 | osteoglycin |
| *Rpl27a* | NM_011975.3 | 0.036 | 1.41 | 1.78 | ribosomal protein L27a |
| *Yod1* | NM_178691.2 | 0.036 | 1.02 | -1.26 | YOD1 OTU deubiquitinating enzyme 1 homologue (S. cerevisiae) |
| *Pcdhgb1* | NM_033574 | 0.036 | 1.13 | 1.22 | protocadherin gamma subfamily B, 1 |
| *Ankrd47* | NM_030697.1 | 0.036 | 1.22 | 1.10 | ankyrin repeat domain 47 |
| *En1* | NM_010133.1 | 0.036 | 1.40 | 1.46 | engrailed 1 |
| *Glrx* | NM_053108.2 | 0.036 | 1.56 | 1.38 | glutaredoxin |
| *Katna1* | NM_011835.1 | 0.036 | 1.28 | 1.57 | katanin p60 (ATPase-containing) subunit A1 |
| *Rps6ka4* | NM_019924.1 | 0.036 | 1.21 | 1.51 | ribosomal protein S6 kinase, polypeptide 4 |
| *Scamp2* | NM_022813.2 | 0.036 | 1.16 | -1.05 | secretory carrier membrane protein 2 |
| *Serf1* | NM_011353.1 | 0.036 | 2.01 | 1.86 | small EDRK-rich factor 1 |
| *Tmem8* | NM_021793.1 | 0.036 | 1.27 | 1.20 | transmembrane protein 8 (five membrane-spanning domains) |
| *Vil2* | NM_009510 | 0.036 | 1.64 | 1.55 | ezrin |
| *Zfp397* | NM_027007.1 | 0.036 | 1.21 | 1.38 | zinc finger protein 397 |
| *Optc* | NM_054076.1 | 0.036 | 1.12 | 1.13 | opticin |
| *Nme5* | NM_080637 | 0.036 | 1.87 | 1.50 | non-metastatic cells 5, protein expressed in (nucleoside-diphosphate kinase) |
| *Usp8* | NM_019729.2 | 0.036 | 1.04 | -1.21 | ubiquitin specific peptidase 8 |
| *Zfp428* | NM_146183.1 | 0.036 | 1.58 | 1.92 | zinc finger protein 428 |
| *Zfp709* | NM_145624.2 | 0.036 | 1.33 | 1.32 | zinc finger protein 709 |
| *Eno3* | NM_007933.2 | 0.036 | 1.83 | 2.17 | enolase 3, beta muscle |
| *Dpp8* | NM_028906.2 | 0.036 | 1.40 | 1.33 | dipeptidylpeptidase 8 |
| *Scg3* | NM_009130.1 | 0.036 | 1.83 | 1.57 | secretogranin III (Scg3), mRNA. |
| *Apg12l* | NM_026217.1 | 0.036 | 1.31 | 1.23 | autophagy-related 12 (yeast) |
| *Fhl1* | NM_010211.1 | 0.036 | 1.28 | 1.36 | four and a half LIM domains 1 |
| *Lasp1* | NM_010688.2 | 0.036 | 1.42 | 1.36 | LIM and SH3 protein 1 |
| *Dctn2* | NM_027151.1 | 0.036 | 1.19 | 1.24 | dynactin 2 |
| *Bard1* | NM_007525.1 | 0.037 | 1.04 | 1.28 | BRCA1 associated RING domain 1 |
| *Prkra* | NM_011871.1 | 0.037 | 1.33 | 1.41 | protein kinase, interferon inducible double stranded RNA dependent activator |
| *Btf3* | NM_145455.1 | 0.037 | 1.81 | 1.95 | basic transcription factor 3 |
| *Pacrg* | XM_128418.1 | 0.037 | 1.76 | 1.35 | Park2 co-regulated |
| *Gucy1a2* | NM_001033322.1 | 0.037 | 1.19 | 1.28 | guanylate cyclase 1, soluble, alpha 2 |
| *Fez1* | NM_183171.1 | 0.037 | 1.60 | 1.51 | fasciculation and elongation protein zeta 1 |
| *Spc18* | NM_019951.1 | 0.037 | 1.34 | 1.40 | SEC11 homolog A (S. cerevisiae) |
| *Csdc2* | NM_145473.1 | 0.037 | 1.31 | 1.10 | cold shock domain containing C2, RNA binding |
| *Fchsd2* | NM_199012.1 | 0.037 | 1.55 | 1.65 | FCH and double SH3 domains 2 |
| *Mtap1b* | NM_008634.1 | 0.037 | 1.75 | 1.67 | microtubule-associated protein 1 B |
| *Rab7l1* | NM_144875.1 | 0.037 | 2.11 | 2.02 | RAB7, member RAS oncogene family-like 1 |
| *Rai14* | NM_030690.2 | 0.037 | 1.10 | 1.38 | retinoic acid induced 14 |
| *Vamp8* | NM_016794.2 | 0.037 | 1.53 | 1.49 | vesicle-associated membrane protein 8 |
| *Sepx1* | NM_013759.1 | 0.037 | 1.63 | 1.58 | selenoprotein X 1 |
| *Bbc3* | NM_133234.1 | 0.037 | 1.41 | 1.27 | Bcl-2 binding component 3 |
| *Alg9* | NM_133981.1 | 0.037 | 1.37 | 1.33 | asparagine-linked glycosylation 9 homolog (yeast, alpha 1,2 mannosyltransferase) |
| *Dcxr* | NM_026428.1 | 0.037 | 1.65 | 1.59 | dicarbonyl L-xylulose reductase |
| *Eln* | NM_007925.2 | 0.037 | 1.62 | 1.46 | elastin |
| *Pfkp* | NM_019703 | 0.037 | 1.13 | 1.14 | phosphofructokinase, platelet |
| *Glud1* | NM_008133.3 | 0.037 | 1.42 | 1.29 | glutamate dehydrogenase 1 |
| *Il17rb* | NM_019583 | 0.037 | 1.68 | 1.58 | interleukin 17 receptor B |
| *Birc5* | NM_009689.1 | 0.037 | 1.41 | 1.40 | baculoviral IAP repeat-containing 5 |
| *Gm2a* | NM_010299.2 | 0.037 | 1.38 | 1.25 | GM2 ganglioside activator protein |
| *Mark2* | NM_007928 | 0.037 | 1.15 | 1.20 | MAP/microtubule affinity-regulating kinase 2 |
| *Ptpn9* | NM_019651 | 0.037 | 1.09 | 1.22 | protein tyrosine phosphatase, non-receptor type 9 |
| *Rfx2* | NM_009056.1 | 0.037 | 1.58 | 1.27 | regulatory factor X, 2 (influences HLA class II expression) |
| *Grit* | NM_177379.2 | 0.037 | 1.63 | 1.63 | Rho GTPase-activating protein |
| *Acta1* | NM_009606.1 | 0.037 | 1.23 | 1.24 | actin, alpha 1, skeletal muscle |
| *Frmd6* | NM_028127.3 | 0.037 | 1.37 | 1.46 | FERM domain containing 6 |
| *Zfp36* | NM_011756.3 | 0.038 | 1.32 | 1.50 | zinc finger protein 36 |
| *Fh1* | NM_010209.1 | 0.038 | 1.37 | 1.29 | fumarate hydratase 1 |
| *Blcap* | NM_016916.2 | 0.038 | 1.22 | 1.58 | bladder cancer associated protein homolog |
| *Ubadc1* | NM_133835.1 | 0.038 | 1.42 | 1.34 | ubiquitin associated domain containing 1 |
| *Traf3ip2* | NM_134000.2 | 0.038 | 1.49 | 1.37 | Traf3 interacting protein 2 |
| *Ripk1* | NM_009068.3 | 0.038 | 1.45 | 1.33 | receptor (TNFRSF)-interacting serine-threonine kinase 1 |
| *Cyp4f14* | NM_022434.1 | 0.038 | 2.48 | 1.99 | cytochrome P450, family 4, subfamily f, polypeptide 14 |
| *Pbk* | NM_023209.1 | 0.038 | 1.46 | 1.34 | PDZ binding kinase |
| *Cdc2a* | NM_007659.2 | 0.038 | 1.62 | 1.78 | cell division cycle 2 homolog A (S. pombe) |
| *Zbtb2* | NM_001033466.1 | 0.038 | 1.01 | 1.35 | zinc finger and BTB domain containing 2 |
| *Exosc4* | NM_175399.2 | 0.038 | 1.16 | -1.32 | exosome component 4 |
| *Ccnd1* | NM_007631.1 | 0.038 | 5.72 | 7.26 | cyclin D1 |
| *Ramp1* | NM_016894.1 | 0.038 | 1.65 | 1.43 | receptor (calcitonin) activity modifying protein 1 |
| *Stard3nl* | NM_024270.1 | 0.038 | 1.53 | 1.39 | STARD3 N-terminal like |
| *Htr2a* | NM_172812.1 | 0.039 | 1.03 | 1.13 | 5-hydroxytryptamine (serotonin) receptor 2A |
| *Ogfrl1* | NM_001081079.1 | 0.039 | 1.41 | 1.41 | opioid growth factor receptor-like 1 |
| *Pik3cb* | NM_029094.1 | 0.039 | 2.07 | 2.21 | phosphatidylinositol 3-kinase, catalytic, beta polypeptide |
| *Mlp* | NM_010807.2 | 0.039 | 1.55 | 1.64 | MARCKS-like 1 |
| *Pdlim7* | NM_026131 | 0.039 | 2.06 | 2.05 | PDZ and LIM domain 7 |
| *Attp* | NM_016855 | 0.039 | 1.20 | 1.43 | signaling molecule ATTP |
| *Lrrk2* | NM_025730.2 | 0.039 | 1.22 | 1.43 | leucine-rich repeat kinase 2 |
| *Rps3a* | NM_016959.2 | 0.039 | 1.41 | 1.73 | ribosomal protein S3a |
| *Ctnnb1* | NM_007614.2 | 0.039 | 1.32 | 1.38 | catenin (cadherin associated protein), beta 1 |
| *Sp9* | NM_001005343.1 | 0.039 | 1.15 | 1.18 | trans-acting transcription factor 9 |
| *Olfr365* | NM_146662.1 | 0.039 | 1.14 | 1.15 | olfactory receptor 365 |
| *Ddah1* | NM_026993 | 0.039 | 1.78 | 1.59 | dimethylarginine dimethylaminohydrolase 1 |
| *Ppm2c* | NM_001033453.1 | 0.039 | 1.86 | 1.52 | protein phosphatase 2C, magnesium dependent, catalytic subunit |
| *Dpp3* | NM_133803.1 | 0.040 | 1.02 | -1.10 | dipeptidylpeptidase 3 |
| *Mboat2* | NM_026037.2 | 0.040 | 1.37 | 1.10 | membrane bound O-acyltransferase domain containing 2 |
| *Gprk6* | NM_001038018.1 | 0.040 | 1.30 | 1.43 | G protein-coupled receptor kinase 6 |
| *Rab33b* | NM_016858.1 | 0.040 | 1.58 | 1.33 | RAB33B, member of RAS oncogene family |
| *Pou5f1* | NM_013633.1 | 0.040 | 1.08 | 1.06 | POU domain, class 5, transcription factor 1 |
| *Nicn1* | NM_025449.2 | 0.040 | 1.58 | 1.45 | nicolin 1 |
| *Pcmt1* | NM_008786.1 | 0.040 | 1.53 | 1.53 | protein-L-isoaspartate (D-aspartate) O-methyltransferase 1 |
| *Dnaic1* | NM_175138.2 | 0.040 | 1.65 | 1.59 | dynein, axonemal, intermediate chain 1 |
| *Dgkg* | NM_138650 | 0.040 | 1.71 | 1.74 | diacylglycerol kinase, gamma |
| *Dbn1* | NM_019813 | 0.040 | 1.38 | 1.40 | drebrin 1 |
| *Rps27* | NM_027015.1 | 0.041 | 1.40 | 1.60 | ribosomal protein S27 |
| *Slamf8* | XM_129596.2 | 0.041 | 1.12 | 1.20 | B lymphocyte activator macrophage expressed |
| *Bcl10* | NM_009740.1 | 0.041 | 1.31 | 1.38 | B-cell leukemia/lymphoma 10 |
| *Cdc42ep4* | NM_020006.1 | 0.041 | 1.72 | 1.39 | CDC42 effector protein (Rho GTPase binding) 4 |
| *Socs7* | NM_138657.1 | 0.041 | 1.06 | 1.15 | suppressor of cytokine signaling 7 |
| *Ckb* | NM_021273 | 0.041 | 1.18 | 1.16 | creatine kinase, brain |
| *Ints3* | NM_145540.2 | 0.041 | 1.28 | 1.44 | integrator complex subunit 3 |
| *Spc25* | NM_025565.1 | 0.042 | 1.43 | 1.52 | SPC25, NDC80 kinetochore complex component, homolog (S. cerevisiae) |
| *Zfp289* | NM_023854.1 | 0.042 | 1.10 | -1.15 | zinc finger protein 289 |
| *Tmprss11a* | NM_001033233.1 | 0.042 | 1.16 | 1.17 | transmembrane protease, serine 11a |
| *Tmem115* | NM_019704 | 0.042 | 1.37 | 1.13 | transmembrane protein 115 |
| *Odf2* | NM_013615 | 0.042 | 1.48 | 1.33 | outer dense fiber of sperm tails 2 |
| *Tmem98* | NM_029537.1 | 0.042 | 1.17 | 1.04 | transmembrane protein 98 |
| *Sfxn1* | NM_027324.2 | 0.042 | 1.31 | 1.26 | sideroflexin 1 |
| *Dlx4* | NM_007867.1 | 0.042 | 1.12 | 1.00 | distal-less homeobox 4 |
| *Cd9* | NM_007657.2 | 0.042 | 1.39 | 1.30 | CD9 antigen |
| *Suclg2* | NM_011507.1 | 0.042 | 1.50 | 1.57 | succinate-Coenzyme A ligase, GDP-forming, beta subunit |
| *Stk11* | NM_011492.1 | 0.042 | 1.36 | 1.19 | serine/threonine kinase 11 |
| *Dync2li1* | NM_172256.1 | 0.042 | 1.02 | -1.18 | dynein cytoplasmic 2 light intermediate chain 1 |
| *Fbxl12* | NM_013911.1 | 0.042 | 1.28 | 1.23 | F-box and leucine-rich repeat protein 12 |
| *Ddt* | NM_010027.1 | 0.042 | 1.34 | 1.41 | D-dopachrome tautomerase |
| *Nme7* | NM_178071.2 | 0.043 | 1.32 | 1.24 | non-metastatic cells 7, protein expressed in |
| *Rcbtb2* | NM_134083.2 | 0.043 | 1.26 | 1.13 | regulator of chromosome condensation (RCC1) and BTB (POZ) domain containing protein 2 |
| *Smad1* | NM_008539.3 | 0.043 | 1.44 | 1.54 | MAD homolog 1 (Drosophila) |
| *Tspyl1* | NM_009433.2 | 0.043 | 1.00 | -1.26 | testis-specific protein, Y-encoded-like 1 |
| *Dbi* | NM_007830.2 | 0.043 | 1.39 | 1.19 | diazepam binding inhibitor |
| *Flna* | XM_289920.2 | 0.043 | 1.32 | 1.78 | filamin, alpha |
| *Ifitm3* | NM_025378.1 | 0.043 | 1.78 | 1.72 | interferon induced transmembrane protein 3 |
| *Lingo2* | NM_175516.2 | 0.043 | 1.26 | 1.16 | leucine rich repeat and Ig domain containing 2 |
| *Cdc20* | NM_023223.1 | 0.043 | 1.14 | 1.14 | cell division cycle 20 homolog (S. cerevisiae) |
| *Tmod2* | NM_001038710.1 | 0.043 | 2.48 | 2.47 | tropomodulin 2 |
| *Cyhr1* | NM_180962.1 | 0.043 | 1.05 | -1.26 | cysteine and histidine rich 1 |
| *Hist1h2be* | NM_178194.2 | 0.043 | 1.73 | 1.46 | histone cluster 1, H2be |
| *Nell2* | NM_016743.1 | 0.043 | 1.03 | 1.20 | NEL-like 2 (chicken) |
| *Rab5b* | NM_011229.1 | 0.043 | 1.24 | 1.04 | RAB5B, member RAS oncogene family |
| *Rps15* | NM_009091.1 | 0.043 | 1.32 | 1.40 | ribosomal protein S15 |
| *Haghl* | NM_026897.1 | 0.043 | 1.45 | 1.12 | hydroxyacylglutathione hydrolase-like |
| *Pgpep1* | NM_023217.2 | 0.043 | 1.28 | 1.00 | pyroglutamyl-peptidase I |
| *Trim28* | NM_011588.1 | 0.043 | 1.24 | 1.34 | tripartite motif protein 28 |
| *Hspa5bp1* | NM_133804.1 | 0.044 | 1.27 | 1.18 | heat shock protein 5 binding protein 1 |
| *Hspb8* | NM_030704.1 | 0.044 | 1.41 | 1.56 | heat shock protein 8 |
| *Ppp1r1a* | NM_021391.2 | 0.044 | 1.58 | 1.47 | protein phosphatase 1, regulatory (inhibitor) subunit 1A |
| *Reck* | NM_016678.1 | 0.044 | 1.34 | 1.59 | reversion-inducing-cysteine-rich protein with kazal motifs |
| *Acot8* | NM_133240.1 | 0.044 | 1.23 | 1.37 | acyl-CoA thioesterase 8 |
| *Serpinb6a* | NM_009254 | 0.044 | 1.59 | 1.44 | serine (or cysteine) peptidase inhibitor, clade B, member 6a |
| *Rps17* | NM_009092.2 | 0.044 | 1.50 | 1.63 | ribosomal protein S17 |
| *Cpsf6* | NM_001013391.1 | 0.044 | 1.07 | 1.39 | cleavage and polyadenylation specific factor 6 |
| *Acad9* | NM_172678.2 | 0.044 | 1.45 | 1.43 | acyl-Coenzyme A dehydrogenase family, member 9 |
| *Mknk2* | NM_021462 | 0.044 | 1.16 | 1.29 | MAP kinase-interacting serine/threonine kinase 2 |
| *Atp6v0e2* | NM_133764.2 | 0.044 | 1.28 | 1.19 | ATPase, H+ transporting, lysosomal V0 subunit E2 |
| *Mapk1ip1* | NM_001045483.1 | 0.044 | 1.05 | -1.24 | mitogen activated protein kinase 1 interacting protein 1 |
| *Olfml1* | NM_172907.2 | 0.044 | 3.70 | 2.30 | olfactomedin-like 1 |
| *Inpp5a* | NM_183144.1 | 0.045 | 1.32 | 1.51 | inositol polyphosphate-5-phosphatase A |
| *Ube2m* | NM_145578.1 | 0.045 | 1.35 | 1.40 | ubiquitin-conjugating enzyme E2M (UBC12 homolog, yeast) |
| *Sp100* | NM_013673.2 | 0.045 | 1.17 | 1.03 | nuclear antigen Sp100 |
| *Reep3* | NM_178606.2 | 0.045 | 1.84 | 1.97 | receptor accessory protein 3 |
| *Atp1b2* | NM_013415.2 | 0.045 | 3.12 | 3.03 | ATPase, Na+/K+ transporting, beta 2 polypeptide |
| *Ap2a2* | NM_007459.2 | 0.045 | 1.22 | 1.53 | adaptor protein complex AP-2, alpha 2 subunit |
| *Fadd* | NM_010175.2 | 0.045 | 1.34 | 1.10 | Fas (TNFRSF6)-associated via death domain |
| *Nudt19* | NM_033080.1 | 0.045 | 1.50 | 1.31 | nudix |
| *Pvrl2* | NM_008990.2 | 0.045 | 1.81 | 1.50 | poliovirus receptor-related 2 |
| *Gamt* | NM_010255.1 | 0.045 | 1.36 | 1.11 | guanidinoacetate methyltransferase |
| *Uchl1* | NM_011670.1 | 0.045 | 1.53 | 1.55 | ubiquitin carboxy-terminal hydrolase L1 |
| *Ccl19* | NM_011888.2 | 0.045 | 1.04 | 1.16 | chemokine (C-C motif) ligand 19 |
| *Etfa* | NM_145615.2 | 0.045 | 1.27 | 1.25 | electron transferring flavoprotein, alpha polypeptide |
| *Zfp297* | NM_020625.2 | 0.045 | 1.19 | -1.45 | zinc finger and BTB domain containing 22 |
| *Mthfd1* | NM_138745.1 | 0.045 | 1.35 | 1.33 | methylenetetrahydrofolate dehydrogenase |
| *H19* | NR_001592.1 | 0.045 | 1.69 | 1.64 | H19 fetal liver mRNA |
| *Vamp4* | NM_016796.2 | 0.045 | 1.06 | -1.40 | vesicle-associated membrane protein 4 |
| *Rapgef3* | NM_144850.1 | 0.046 | 1.79 | 1.64 | Rap guanine nucleotide exchange factor (GEF) 3 |
| *Zfp313* | NM_030743.3 | 0.046 | 1.20 | 1.38 | zinc finger protein 313 |
| *Oplah* | NM_153122.1 | 0.046 | 1.58 | 1.41 | 5-oxoprolinase (ATP-hydrolysing) |
| *Slc7a6* | NM_178798.2 | 0.046 | 1.11 | 1.25 | solute carrier family 7 (cationic amino acid transporter, y+ system), member 6 |
| *Thsd6* | NM_025629.1 | 0.046 | 1.54 | 1.53 | ADAMTS-like 5 |
| *Adam12* | NM_007400 | 0.046 | 2.08 | 2.58 | disintegrin and metallopeptidase domain 12 |
| *Pcyox1l* | NM_172832.1 | 0.046 | 1.49 | 1.41 | prenylcysteine oxidase 1 like |
| *Snx10* | NM_028035.2 | 0.046 | 1.27 | 1.33 | sorting nexin 10 |
| *Tubd1* | NM_019756.1 | 0.046 | 1.19 | 1.13 | tubulin, delta 1 |
| *Ncaph2* | NM_025795.2 | 0.046 | 1.20 | 1.05 | non-SMC condensin II complex, subunit H2 |
| *Mbp* | NM_001025245.1 | 0.046 | 1.39 | 1.28 | myelin basic protein |
| *Prdx2* | NM_011563.2 | 0.046 | 1.17 | -1.09 | peroxiredoxin 2 |
| *Smad3* | NM_016769.2 | 0.047 | 1.21 | 1.17 | MAD homolog 3 (Drosophila) |
| *H1f0* | NM_008197.2 | 0.047 | 1.37 | -1.02 | H1 histone family, member 0 |
| *Phlda1* | NM_009344.1 | 0.047 | 1.01 | 3.62 | pleckstrin homology-like domain, family A, member 1 |
| *Paqr9* | NM_198414.2 | 0.047 | 1.19 | 1.40 | progestin and adipoQ receptor family member IX |
| *Cox7a2* | NM_009945.2 | 0.047 | 1.19 | 1.25 | cytochrome c oxidase, subunit VIIa 2 |
| *Tuba1a* | NM_011653.1 | 0.047 | 1.37 | 1.27 | tubulin, alpha 1A |
| *Smpd3* | NM_021491.2 | 0.047 | 1.58 | 1.79 | sphingomyelin phosphodiesterase 3 |
| *Nde1* | NM_023317 | 0.047 | 1.52 | 1.51 | nuclear distribution gene E homolog 1 (A nidulans) |
| *Gnat1* | NM_008140.2 | 0.048 | 1.56 | 1.27 | guanine nucleotide binding protein, alpha transducing 1 |
| *Ap2s1* | NM_198613.1 | 0.048 | 1.36 | 1.33 | adaptor-related protein complex 2, sigma 1 subunit |
| *Thrap5* | NM_198107.1 | 0.048 | 1.83 | 1.62 | mediator complex subunit 16 |
| *Nek6* | NM_021606.2 | 0.048 | 1.28 | 1.41 | NIMA (never in mitosis gene a)-related expressed kinase 6 |
| *Klf3* | NM_008453 | 0.048 | 1.24 | 1.58 | Kruppel-like factor 3 |
| *Dab1* | NM_010014.1 | 0.048 | 1.25 | 1.49 | disabled homolog 1 |
| *Ercc2* | NM_146182.1 | 0.048 | 1.59 | 1.40 | kinesin light chain 3 |
| *Helb* | NM_080446.1 | 0.048 | 1.09 | -1.14 | helicase (DNA) B |
| *Tpi1* | NM_009415.1 | 0.048 | 1.32 | 1.30 | triosephosphate isomerase 1 |
| *Atg5* | NM_053069.2 | 0.048 | 1.19 | 1.42 | autophagy-related 5 (yeast) |
| *Il6st* | NM_010560 | 0.048 | 1.93 | 1.98 | interleukin 6 signal transducer |
| *Kbtbd7* | NM_001024135.1 | 0.048 | 1.36 | 1.23 | kelch repeat and BTB (POZ) domain containing 7 |
| *Cdc37* | NM_016742.2 | 0.049 | 1.21 | 1.04 | cell division cycle 37 homolog (S. cerevisiae) |
| *Esco2* | NM_028039.1 | 0.049 | 1.26 | 1.09 | establishment of cohesion 1 homolog 2 (S. cerevisiae) |
| *Shrm* | NM_015756 | 0.049 | 1.10 | 1.17 | shroom family member 3 |
| *Rps19* | NM_023133.1 | 0.049 | 1.87 | 2.07 | ribosomal protein S19 |
| *Rbm26* | NM_134077.4 | 0.049 | 1.10 | 1.64 | RNA binding motif protein 26 |
| *Mobkl2a* | NM_172457.2 | 0.049 | 1.18 | 1.14 | MOB1, Mps One Binder kinase activator-like 2A (yeast) |
| *Cldn11* | NM_008770.1 | 0.049 | 2.13 | 1.90 | claudin 11 |
| *Prosapip1* | NM_197945.2 | 0.049 | 1.26 | -1.07 | ProSAPiP1 protein |
| *Zfp37* | NM_009554.3 | 0.049 | 1.21 | -1.05 | zinc finger protein 37 |
| *Anxa11* | NM_013469.1 | 0.049 | 1.40 | 1.25 | annexin A11 |
| *Rarb* | NM_011243 | 0.049 | 1.25 | 1.28 | retinoic acid receptor, beta |
| *Creb3l2* | NM_178661.2 | 0.049 | 1.11 | 1.26 | cAMP responsive element binding protein 3-like 2 |
| *Mras* | NM_008624.2 | 0.049 | 1.62 | 1.80 | muscle and microspikes RAS |
| *Drp2* | NM_010078.1 | 0.049 | 1.74 | 1.68 | dystrophin related protein 2 |
| *Rsu1* | NM_009105 | 0.049 | 1.44 | 1.42 | Ras suppressor protein 1 |
| *Fancc* | NM_001042673.1 | 0.049 | 1.34 | 1.23 | Fanconi anemia, complementation group C |
| *Zfp30* | NM_013705.1 | 0.049 | 1.37 | 1.24 | zinc finger protein 30 |

Table of genes significantly induced ethanol (E, 60 mM, 1h) and heat stress (H, 42°C, 1). Data were obtained by hybridization of seven biologically independent samples with treatments performed at least in duplicate. The differences in gene expressions were determined using ANOVA post-hoc adjusted by Tukey test (P<0.05); multiple hypothesis testing adjustment were made using the Benjamini–Hochberg method at a false discovery rate (FDR) of less than 0.05.
